# CD11c^+^ CD8 T cells cause IFN-**γ**–dependent autoimmune neuroinflammation that is restrained by PD-1 signaling

**DOI:** 10.1172/jci.insight.179789

**Published:** 2026-05-22

**Authors:** Daniel Hwang, Gholamreza Azizi, Larissa Lumi Watanabe Ishikawa, Maryam Seyedsadr, Arin Cox, Soohwa Jang, Ezgi Kasimoglu, Abdolmohamad Rostami, Guang-Xian Zhang, Bogoljub Ciric

**Affiliations:** 1 Department of Neurology, Jefferson Hospital for Neuroscience, Thomas Jefferson University, Philadelphia, Pennsylvania, USA.; 2 Comparative Pathology Core, School of Veterinary Medicine, University of Pennsylvania, Philadelphia, Pennsylvania, USA.

**Keywords:** Autoimmunity, Neuroscience, Mouse models, Multiple sclerosis, T cells

## Abstract

In multiple sclerosis (MS) lesions, CD8 T cells outnumber CD4 T cells, suggesting that they contribute to MS pathology. However, little is known about the role of CD8 T cells in MS, partly due to the prevalent use of experimental autoimmune encephalomyelitis (EAE) models mediated by CD4 T cells, which have limited involvement of CD8 T cells. Importantly, MS and EAE differ in both their distribution of CNS lesions and neurologic deficits, indicating differences in CNS inflammation. MS lesions are more commonly found in the brain, whereas EAE lesions are more frequent in the spinal cord. Additionally, neurologic deficits in MS rarely parallel the ascending paralysis typical for CD4 T cell–mediated EAE (CD4-EAE). In contrast, CD8-EAE models suggest that CD8 T cells preferentially cause brain inflammation; however, little is known about how brain and spinal cord inflammation may differ, or how CD8 T cells contribute to these differences. We have established an adoptive CD8-EAE mouse model characterized by brain-centered inflammation, ataxia, and weight loss. CNS inflammation in the brain and spinal cord differed in immune cell numbers, cellular composition, and inflammatory signatures. CD8-EAE could be suppressed by blocking IFN-γ, and exacerbated by blocking PD-1, with concomitant changes in the numbers of CNS-infiltrating monocytes. Most CD8 T cells in the CNS were CD11c^+^, suggesting that they are the pathogenic subset. We describe a robust CD8-EAE model, identify differences between brain and spinal cord inflammation, and characterize mechanisms that control CD8 T cell–mediated neuroinflammation, thereby furthering understanding of EAE and MS.

## Abstract 2

CD8 T cells are understudied in MS due to a lack of suitable animal models. We developed a CD8 T cell dependent model of MS.

## Introduction

Multiple sclerosis (MS) is an autoimmune disease characterized by demyelinated lesions in the brain and, to a lesser extent, in the spinal cord (1). Active MS lesions are laden with immune cells, including monocytic cells, microglia, B cells, and both CD4 and CD8 T cells (2). Among T cells in MS lesions, CD8 T cells are typically twice as numerous as CD4 T cells (2). This is in stark contrast to conventional experimental autoimmune encephalomyelitis (EAE) models of MS, which all depend on CD4 T cells to initiate and sustain disease processes, while CD8 T cells are hardly present in EAE lesions and are dispensable for disease development (3). The exclusive use of CD4 T cell–mediated EAE (CD4-EAE) models in the development of MS therapeutics has led to a bias toward targeting CD4 T cell responses in MS. However, growing evidence suggests that CD8 T cells also contribute to MS pathology. Clinical trials in relapsing-remitting MS (RR-MS) targeting mainly CD4 T cell responses (anti-CD4 and anti–IL-12/IL-23p40 mAbs; refs. 4, 5) failed to show clinical benefit, indicating that other cells, potentially CD8 T cells, can drive the disease. There is also more direct evidence of disease activity resulting from CD8 T cells, including that neuroantigen-specific, clonally expanded CD8 T cells with highly differentiated phenotype are more frequent in patients with MS than in healthy individuals (6). Moreover, CD8 T cells in MS lesions express proinflammatory cytokines (e.g., IFN-γ, IL-17A, GM-CSF) (7) with similar frequency as CD4 T cells, further supporting the possibility that CD8 T cells in MS play a pathogenic role. Clinical and histological features of MS and EAE also differ in ways that could be attributed to pathogenic CD8 T cells. Most MS lesions are found in the brain, not the spinal cord (1), causing an array of neurological deficits, including impaired coordination and sensory defects (8). This is reminiscent of findings by several seminal EAE studies showing that pathogenic myelin-specific CD8 T cells cause brain-centered inflammation, and impaired movement and coordination (9, 10). This is decidedly different than in CD4-EAE models, in which lesions are predominantly in the spinal cord, with lesser involvement of the brain, and disease is characterized by ascending paralysis (11, 12). These observations suggest that pathogenic mechanisms central to the clinical deficits in MS are not recapitulated in CD4-EAE models, despite their near ubiquity in MS research. Moreover, notable clinical similarities to MS found in CD8-EAE studies strongly support the notion that CD8 T cells play a significant pathogenic role in MS.

The dearth of knowledge on CD8 T cells in MS/EAE could also be attributed to the lack of simple and reliable animal models in which CD8 T cells play a central role in causing robust CNS autoimmunity. Existing CD8-EAE models suffer from various experimental caveats, including reliance on CD4 T cells to induce disease (13, 14) or requiring radiation to render recipient mice susceptible to disease (10). Other caveats include the transfer of pathogenic CD8 T cells directly into the CNS, thereby bypassing the need to cross the blood-brain barrier (BBB) (10), or the use of vaccinia viruses to trigger CNS autoimmune disease (9). Nevertheless, the insights from these studies have been highly valuable in revealing potential functions of CD8 T cells in MS. Myelin basic protein–specific (MBP-specific) CD8 T cells from C3HeB/FeJ mice were shown to cause primarily brain lesions and a loss of coordinated movement that is referred to as atypical EAE (10). Later, the T cell receptor (TCR) from an MBP_79-87_-specific CD8 T cell clone, named 8.8, due to expression of Vβ8 and Vα8 TCR, was used to generate TCR transgenic 8.8 mice (9). These mice are largely tolerant of MBP_79-87_ (9) but can develop spontaneous disease later in life (13). The tolerance against MBP_79-87_ in 8.8 mice could be broken by infection with vaccinia virus, via a mechanism that involves spontaneous coexpression of TCRs specific for both vaccinia antigens and MBP_79-87_ on a subset of 8.8 CD8 T cells (9). More recently, it was shown that 8.8 CD8 T cells could modify CD4-EAE by directing inflammation toward the brain, resulting in a higher incidence of atypical EAE (14). In these studies, however, CD4 T cells can obscure the pathogenic mechanisms of CD8 T cells. For example, in early studies with MBP_79-87_-specific CD8 T cells, disease was IFN-γ–dependent, whereas IFN-γ was dispensable in CD4-EAE mice that received 8.8 CD8 T cells (14). Thus, 8.8 mice have been used in several studies as a source of in vivo developed, myelin-reactive T cells, but so far, the development of disease has been dependent on other sensitizing factors, including viral infection, irradiation, and intrathecal transfer (9, 10).

In the present study, we have developed a simple and reliable CD8-EAE model that eliminates the need for irradiation of recipient mice, pertussis toxin injection, intrathecal transfer of CD8 T cells, viral infection, cotransfer of CD4 T cells, or transgenic pseudoautoantigens, which are used in other CD8-EAE models. Our CD8-EAE model is based on the transfer of in vitro–activated 8.8 T cells into naive recipient mice, which then develop ataxia and weight loss, rather than ascending paralysis. This coincided with mostly brain inflammation, although the spinal cord was also affected. Interestingly, most of the transferred 8.8 CD8 T cells became CD11c^+^, a phenotype not previously described in EAE or MS. Disease reproducibly began only several days after the transfer of 8.8 CD8 T cells and was acute and monophasic, unless the mice died at the peak of the disease. Blocking PD-1 in recipient mice or inducing a memory phenotype in 8.8 CD8 T cells prior to transfer exacerbated the disease. Lastly, the transfer of 8.8 CD8 T cells led to an influx of monocytes into the brain and spinal cord, which can be blocked by treatment with anti–IFN-γ mAb, thereby suppressing the disease.

## Results

### Adoptive transfer of 8.8 Tc1 cells causes EAE-like disease characterized by ataxia and weight loss.

**Figure 1:**
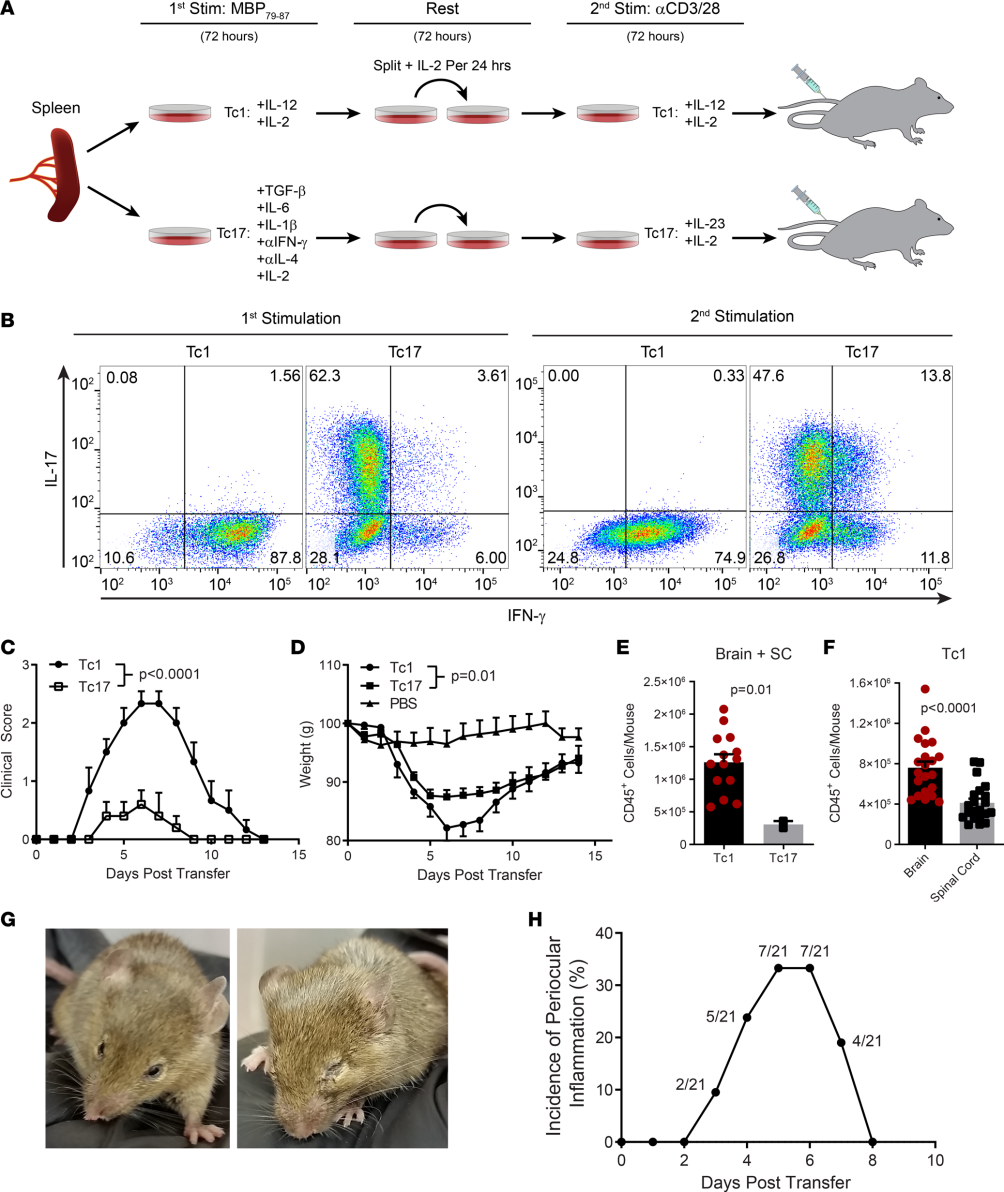
Adoptive transfer of 8.8 Tc1 and Tc17 cells induces autoimmune neuroinflammation. (**A**) Splenocytes from 8.8 mice were stimulated with MBP_79-87_ for 3 days in either Tc1-, or Tc17-polarizing conditions. Tc17 condition: TGF-β (10 ng/mL), IL-6 (50 ng/mL), IL-1β (10 ng/mL), anti–IFN-γ (10 μg/mL), and anti–IL-4 (10 μg/mL). Tc1 condition: IL-12 (20 ng/mL) and IL-2 (5–10 ng/mL). After 72 hours, cells were rested in either 2 ng/mL IL-2 for Tc17 polarization or 5–10 ng/mL IL-2 for Tc1 polarization. Each day, cells were split and replated in fresh media supplemented with IL-2 at concentrations indicated above. After 3 days of rest, cells were reactivated for 2.5 days with plate-bound anti-CD3 and anti-CD28 in the presence of IL-23 (10 ng/mL) and IL-2 (2 ng/mL) for Tc17 cells, and IL-12 (2 ng/mL) and IL-2 (5-10 ng/mL) for Tc1 cells. For CD8-EAE induction, CD8^+^ cells were purified by negative selection using MACS beads, and 2 × 10^7^ cells were transferred to recipient mice via tail vein injection. (**B**) Flow cytometry plots showing Tc1- and Tc17-polarized 8.8 CD8^+^ T cells stained for IL-17A and IFN-γ after first and second activation. (**C**) In total, 2 × 10^7^ MACS-purified CD8^+^ T cells and 0.2–0.4 μg recombinant murine IL-2 were i.v. transferred to naive recipient mice. Clinical course for CD8-EAE (*n* = 5–7 mice per group). Mice from 2 independent experiments in which all animals developed disease are shown. (**D**) Change in weight during CD8-EAE. Significance was determined by 2-way ANOVA. (**E**) Number of CD45^+^ cells isolated from the CNS (brain + spinal cord). (**F**) Number of CD45^+^ cells isolated from the brain or spinal cord. Significance was determined by a *t* test. (**G**) Photos of inflamed eyes during Tc1-mediated CD8-EAE. (**H**) Incidence of periocular inflammation over the course of CD8-EAE (*n* = 21, pooled from 5 independent experiments).

In adoptive CD4-EAE, both Th1 and Th17 cells can cause disease (15). In MS lesions, both CD4 and CD8 T cells produce IFN-γ and IL-17A (7), suggesting that both T cell subsets are pathogenic. Thus, we tested whether Tc1 and Tc17 cells could induce CNS autoimmunity. We polarized 8.8 CD8 T cells into either the Tc1 or Tc17 lineage and expanded them to large numbers (Figure 1, A and B, Supplemental Figure 1C). Tc1 cells uniformly produced IFN-γ, while Tc17-polarized cells produced IL-17A, IFN-γ, or both cytokines. Adoptive transfer of Tc1 or Tc17 cells into naive recipients resulted in an acute, monophasic disease characterized by severe ataxia and rapid weight loss (Figure 1, C and D, and Supplemental Video 1; supplemental material available online with this article; https://doi.org/10.1172/jci.insight.179789DS1). Tc1 cells caused more severe clinical disease, weight loss, and CNS inflammation than Tc17 cells (Figure 1, C–E). Mice transferred with Tc1 cells had greater numbers of CD45+ cells in the brain versus the spinal cord (Figure 1F). Unlike the ascending paralyses characteristic of CD4-EAE (1), mice that developed CD8-EAE retained the ability to move their tails and limbs, but they lost coordinated movement to a varying degree, including loss of the ability to right themselves. At the disease peak, some mice became moribund or died (Supplemental Video 1) but those that survived always fully recovered. Interestingly, one-third of the mice also developed periocular inflammation, which also followed a monophasic disease course (Figure 1, G and H). Notably, Tc1 cells activated in vitro once could induce the disease in recipient mice (Supplemental Figure 1, A and B), similar to twice-activated Tc1 cells (Figure 1, C and D). As reported in some studies on adoptive CD4-EAE (16), the administration of pertussis toxin to recipient mice resulted in less severe CD8-EAE (Supplemental Figure 1, A and B). These data show that both Tc1 and Tc17 8.8 cells can induce disease, starting only several days after transfer into naive recipient mice. In contrast to Th1 and Th17 cells, Tc17 cells were notably less pathogenic than Tc1 cells, which prompted us to continue our studies using Tc1 cells. Overall, 8.8 CD8 T cells cause prominent inflammation in both the brain and spinal cord, which clinically manifests distinctly from CD4-EAE. The disease is self-resolving, as mice that survive the disease fully recover. The monophasic nature of CD8 T cell–mediated neuroinflammation has been largely overlooked to date.

### Tc1 cells cause distinct inflammation in the brain and spinal cord.

**Figure 2:**
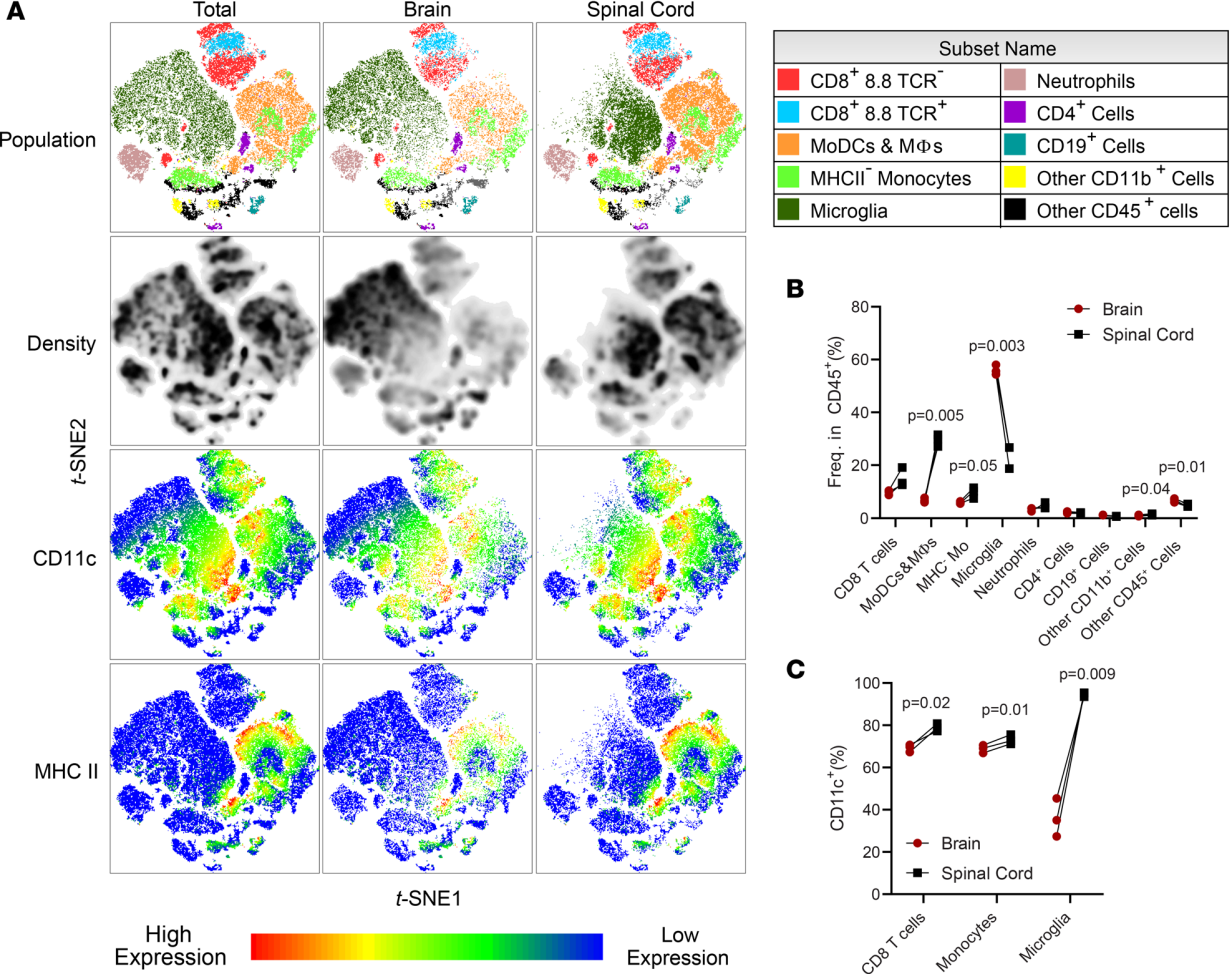
Tc1 cells cause distinct inflammation in the brain and spinal cord. (**A**) Splenocytes from 8.8 mice were activated with MBP_79-87_ for 3 days in Tc1 condition, followed by 3 days of expansion in IL-2 and CD8 T cell purification by MACS negative selection. In total, 2 × 10^7^ cells were transferred i.v. into recipient mice. Mice were sacrificed 7 days later, and the brains and spinal cords were analyzed separately by flow cytometry. Data from 1 (*n* = 3) of 2 experiments with similar results are shown. CD45^+^ cells from the CNS of recipient mice were clustered using t*-*SNE, and populations are colored according to manual gating. CD8 T cells were defined as CD45^hi^CD11b^–^CD8^+^, with 8.8 TCR^+^ and 8.8 TCR^–^ cells being defined as TCR Vα8.3^+^Vβ8.1/8.2^+^ and TCR Vα8.3^–^Vβ8.1/8.2^–^, respectively. MoDCs and MFs were defined as CD45^hi^CD11b^+^Ly6G^lo/–^Ly6C^+^MHCII^+^, microglia as CD45^lo^CD11b^+^, neutrophils as CD45^+^ CD11b^+^Ly6G^hi^, and CD4^+^ cells as CD45^hi^CD11b^–^CD4^+^. B cells were CD45^hi^CD11b^–^CD19^+^. Other CD11b^+^ cells were CD45^hi^CD11b^+^CD4^–^CD8^–^Ly6G^–^Ly6C^–^. Other CD45^+^ cells were CD45^+^CD4^–^CD8^–^CD11b^–^CD19^–^. (**B**) Quantification of frequency of populations among CD45^+^ cells from **A**. (**C**) Frequency of CD11c^+^ cells among CD45^hi^CD8^+^ T cells, CD45^hi^CD11b^+^Ly6G^–^Ly6C^+^ monocytes, and CD45^lo^CD11b^+^ microglia.

**Figure 3:**
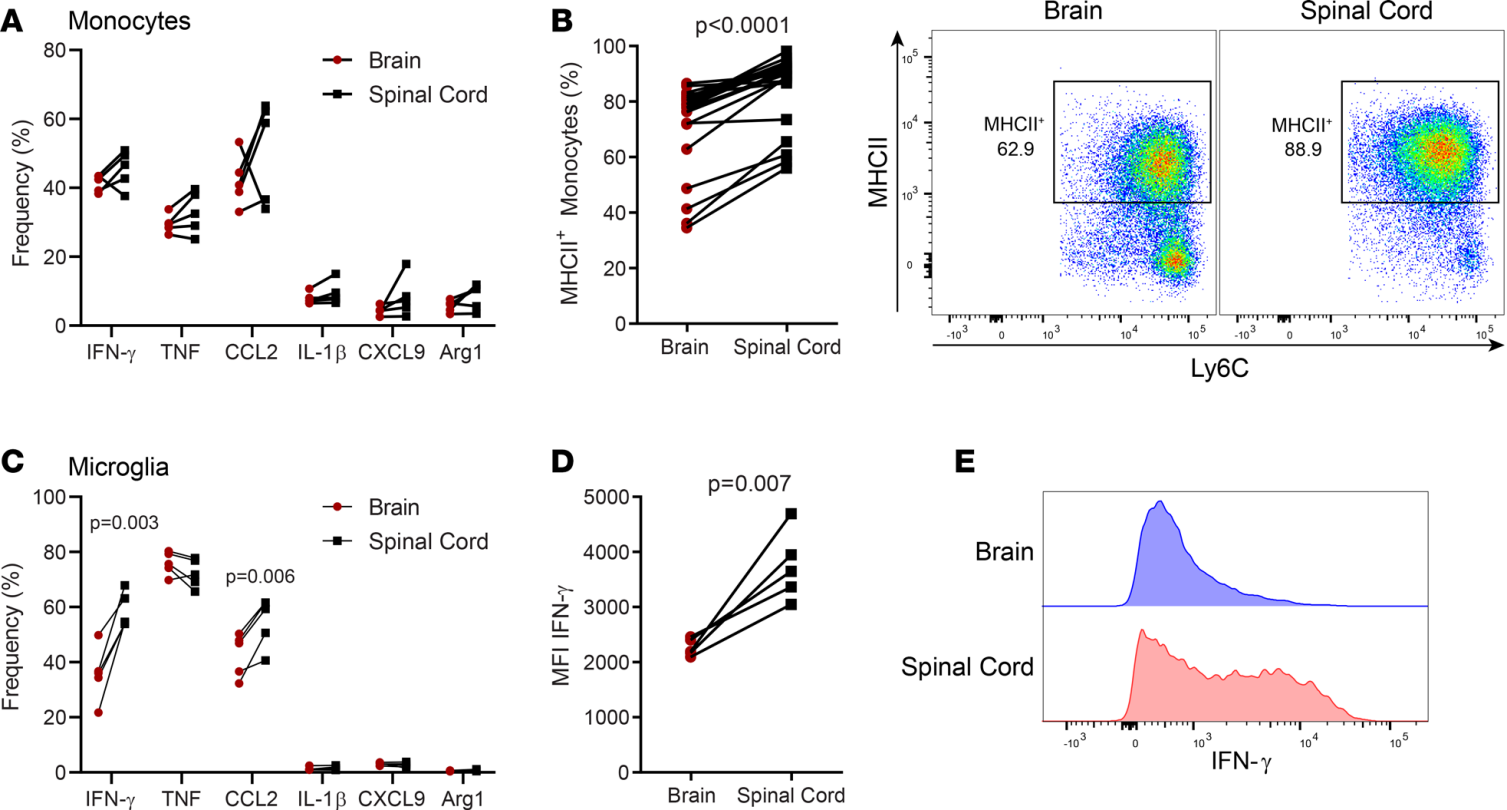
Myeloid cells exhibit distinct inflammatory profiles in the brains and spinal cords of mice with CD8-EAE. (**A**) Expression of indicated cytokines and chemokines amongst monocytes from cells treated with PMA/ionomycin/golgiplug. (**B**) Frequency of MHCII^+^ cells among monocytes. (**C**) Expression of indicated cytokines and chemokines amongst microglia treated with PMA/ionomycin/golgiplug. (**D**) Median fluorescence intensity (MFI) of IFN-γ^+^ microglia. (**E**) Histograms depicting IFN-γ expression in microglia. Significance was determined by a paired *t* test.

To characterize CNS inflammation mediated by 8.8 Tc1 cells, we profiled immune cells from the brain and spinal cord at the peak of the disease. CD45^+^ cells from the CNS were virtually all CNS-resident immune cells and immune cells infiltrated into CNS tissue that were not contaminated with cells from the blood due to incomplete perfusion (Supplemental Figure 2A). Among CD45^+^ cells from the CNS, the predominant cell types were CD8 T cells, monocytes and monocyte-derived cells, and microglia (Figure 2A). CD8 T cells comprised most of lymphocytes in the CNS, with only 1%–3% of immune cells being CD4^+^ T cells and CD19^+^ B cells (Figure 2B). Over 95% of CD8^+^ cells were CD3^+^, and nearly all CD45^lo^CD11b^+^ cells were Sall1^+^ microglia (Supplemental Figure 2, B–D). There were notable differences in immune cell composition and their phenotypes between the brain and spinal cord. Monocytic DCs (MoDCs) and MFs, which are highly inflammatory myeloid cells in CD4-EAE models (16), were present in higher frequencies in the spinal cord compared with the brain. In the brain, the majority of myeloid cells were microglia (Figure 2B). Myeloid cells in the spinal cord appeared to be more activated, with a higher frequency of CD11c^+^ and MHCII^+^ monocytes, and nearly all microglia expressed CD11c (Figure 2C and Figure 3B). Notably, the majority of CD8 T cells also expressed CD11c (Figure 2C). In both the brain and spinal cord, there were similar frequencies of monocytes expressing IFN-γ, TNF, and CCL2, but only a few monocytes expressed IL-1β, CXCL9, and arginase 1 (Figure 3A). Monocytes from the brain and spinal cord had a similar frequency of IFN-γ expression, but lower MFI for IFN-γ from the spinal cord (Figure 3A and Supplemental Figure 2, E and F). A higher frequency of microglia from the spinal cord expressed IFN-γ, with higher MFI for IFN-γ than in the brain (Figure 3, C–E). Interestingly, a higher frequency of microglia expressed CCL2 in the spinal cord (Figure 3C), suggesting that CCL2 secreted by microglia attracts more monocytes in the spinal cord. These data suggest that, although overall infiltrating immune cell numbers are lower in the spinal cord, both brain and spinal cord inflammation are likely relevant to pathology in CD8-EAE.

### CD8 T cells in the CNS are predominantly CD11c^+^ and have an inflammatory phenotype.

**Figure 4:**
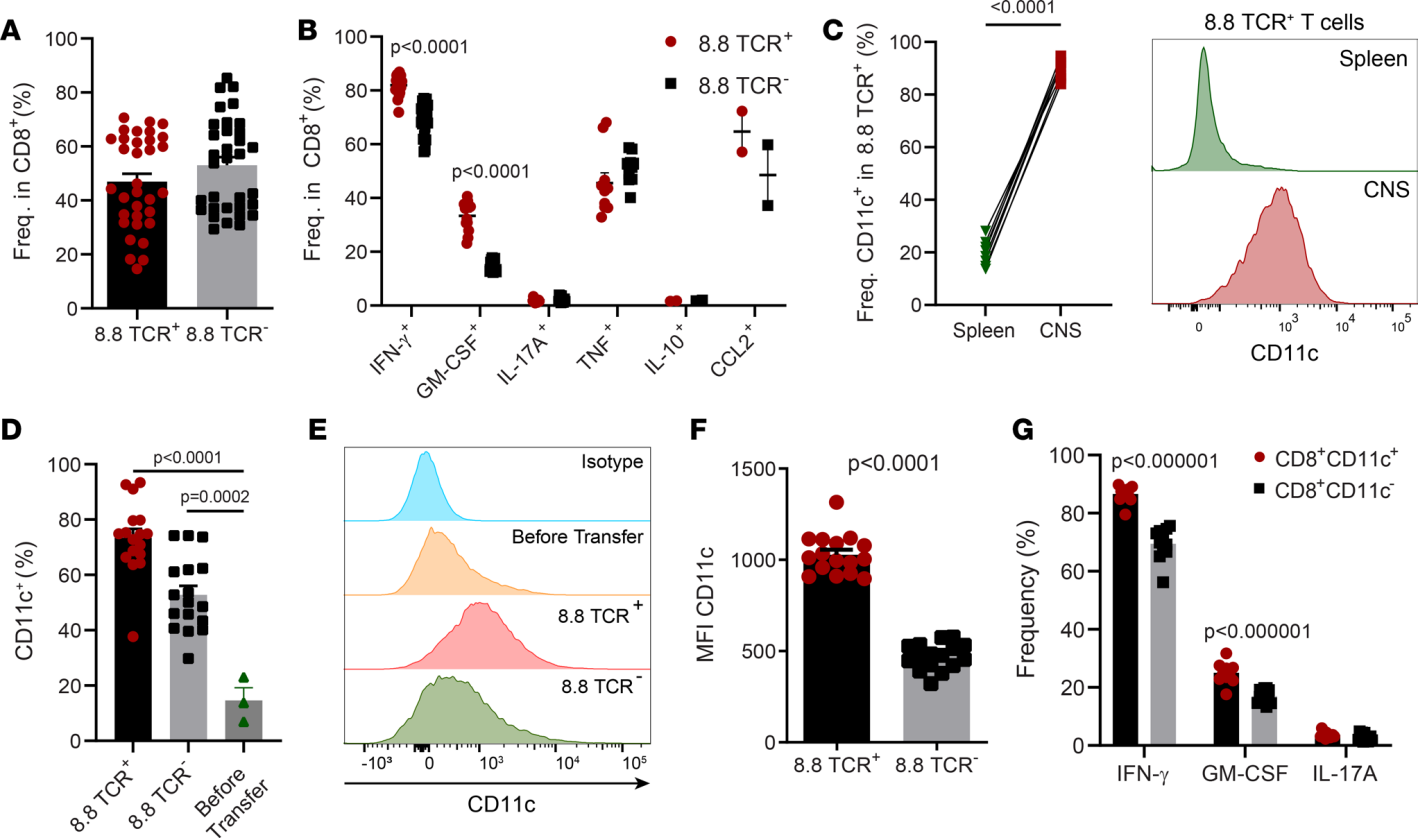
CD8 T cells in the CNS during CD8-EAE are predominantly CD11c^+^ with a proinflammatory phenotype. CD8-EAE was induced by i.v. transfer of 2 × 10^7^ Tc1 cells, and mice were sacrificed after 7 days. Brain and spinal cord cells were combined and analyzed by flow cytometry. (**A**) Frequency of 8.8 TCR^+^ cells (TCR Vβ8.1/8.2^+^ TCR Vα8.3^+^) and 8.8 TCR^–^ cells among CD8^+^ cells. *n* = 33 mice, from 5 experiments. (**B**) Frequency of IFN-γ^+^, GM-CSF^+^, IL-17A^+^, TNF^+^, IL-10^+^, CCL2^+^ cells among 8.8 TCR^+^ CD8^+^ cells and 8.8 TCR^–^ CD8^+^ cells (*n* = 2–22 mice) with data pooled from multiple experiments in all cases except for CCL2. (**C**) Frequency of CD11c^+^ cells among 8.8 TCR^+^CD8^+^ cells in the spleen versus CNS of the same mouse (*n* = 8 mice per group). Significance determined by paired *t* test. Representative flow cytometry plots are shown. (**D**) Frequency of CD11c^+^ cells among 8.8 TCR^+^CD8^+^ cells and 8.8 TCR^–^ CD8^+^ cells in the CNS (*n* = 17 from 3 experiments) and in vitro before transfer (*n* = 3 independent cultures). (**E**) Histograms depicting expression of CD11c by CD8^+^ cells in the CNS and from in vitro cultures. (**F**) MFI of CD11c on 8.8 TCR^+^ and 8.8 TCR^–^ CD8^+^ cells in the CNS (*n* = 16 from 2 experiments). (**G**) Frequency of IFN-γ^+^, GM-CSF^+^, and IL-17A^+^ cells among CD11c^+^CD8^+^ and CD11c^–^CD8^+^ cells (*n* = 12 mice).

We characterized the phenotype of CD8 T cells in the CNS during CD8-EAE. Approximately half of the CD8 T cells did not express transgenic 8.8 TCR (Figure 4A). It is unclear whether these 8.8 TCR^–^ CD8 T cells were endogenous or transferred 8.8 CD8 T cells that had downregulated expression of their 8.8 TCR, but a portion of 8.8 TCR^–^ cells expressed either TCR Vβ8 or Vα8 chain, indicating that these cells derive from the host. Most CD8 T cells in the CNS had Tc1 phenotype, with over 80% of 8.8 TCR^+^ cells and approximately 70% of 8.8 TCR^–^ cells expressing IFN-γ (Figure 4B). CD8 T cells also expressed GM-CSF, TNF, and CCL2, but 8.8 TCR^–^ cells had a lower frequency of GM-CSF expression than 8.8 TCR^+^ cells. Unexpectedly, large proportions of CD8 T cells in the CNS, both 8.8 TCR^+^ and 8.8 TCR^–^, expressed CD11c (Figure 4C). Approximately 80% of 8.8 TCR^+^ cells were CD11c^+^, which is over a 4-fold enrichment in CD11c^+^ cells when compared with their frequency in the spleen or prior to transfer into recipient mice (Figure 4, C and D). The 8.8 TCR cells had a lower frequency of expression and MFI for CD11c than 8.8 TCR^+^ cells (Figure 4, E and F). Consistent with reports that CD11c^+^ CD8 T cells have enhanced effector function, these cells exhibited higher frequencies of IFN-γ and GM-CSF expression than CD11c^–^ cells (Figure 4G).

**Figure 5:**
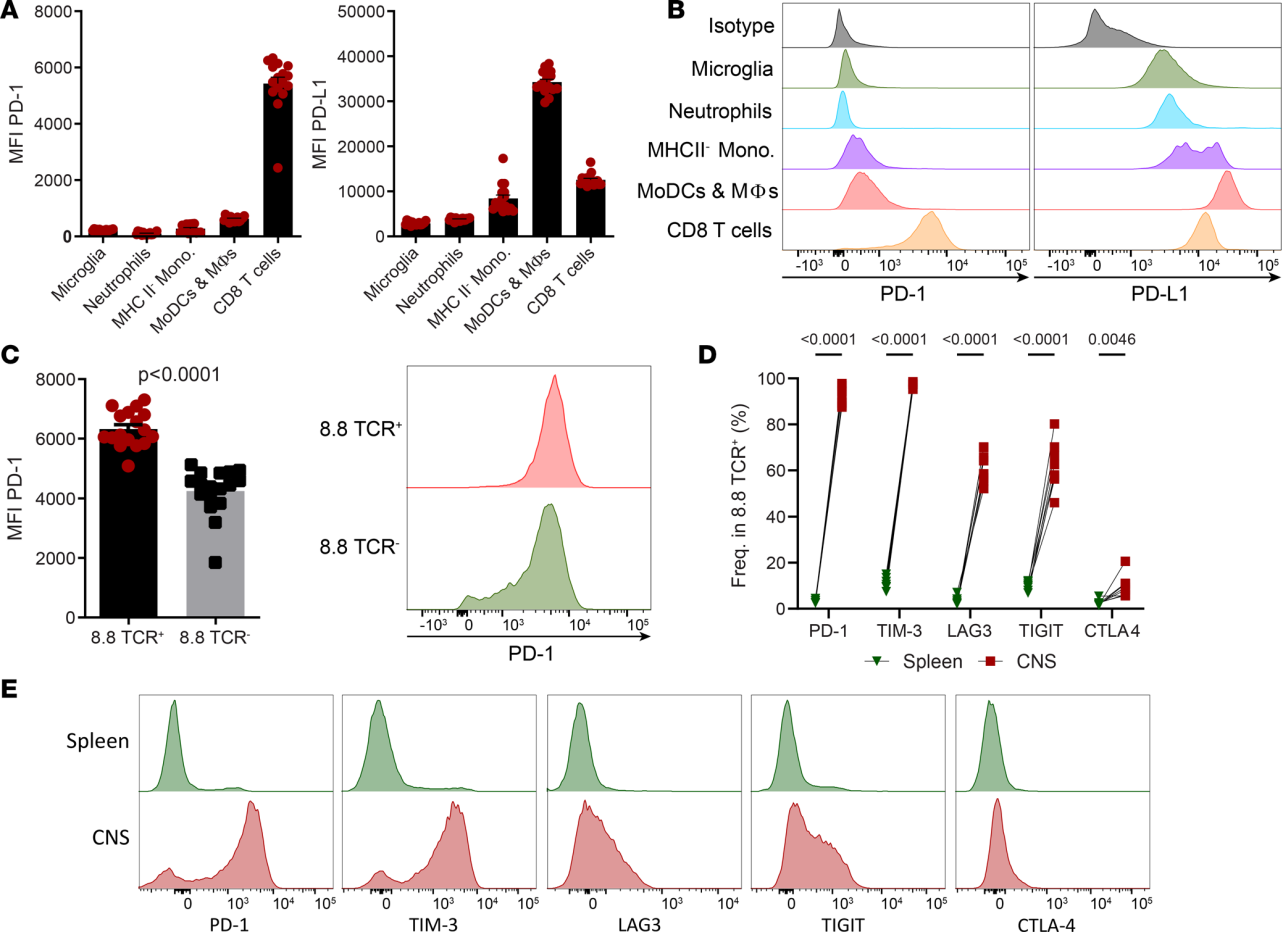
CD8 T cells in the CNS of mice with CD8 EAE express high levels of checkpoint inhibition molecules. (**A**) MFI of PD-L1 and PD-1 among indicated cell populations. (**B**) Histograms depicting expression of PD-1 and PD-L1 by microglia, neutrophils, MHC^–^ monocytes, MoDCs, and MFs, and CD8 T cells. (**C**) MFI for PD-1 among 8.8 TCR^+^ and 8.8 TCR^–^ CD8^+^ T cells and histograms depicting PD-1 expression. For **A**–**C**, *n* = 16 mice from 2 experiments. Significance was determined by an unpaired *t* test. (**D**) Frequency of PD-1, TIM-3, LAG3, TIGIT, and CTLA-4 expressing cells among 8.8 TCR^+^ CD8^+^ cells in spleen and CNS. Significance was calculated by 2-way ANOVA followed by Šídák’s multiple comparisons test. *n* = 8 mice. (**E**) Histograms depicting expression of PD-1, TIM-3, LAG3, TIGIT, and CTLA-4 expressing cells among 8.8 TCR^+^CD8^+^ cells.

Our CD8-EAE model has a monophasic disease course. A possible explanation for the self-resolving inflammation is inhibition of CD8 T cell function by checkpoint inhibitor molecules. Terminal effector phenotypes are typically characterized by high PD-1 expression on CD8^+^ T cells, accompanied by concurrent expression of PD-L1 on myeloid cells at the site of inflammation. To assess if this was the case in our model, we stained for PD-1 and PD-L1 on CNS immune cells during CD8-EAE. PD-1 was abundantly expressed on all CD8 T cells, while myeloid cells expressed little PD-1 (Figure 5, A and B). Among CD45^+^ cells, MoDCs and MFs expressed the highest levels of PD-L1, but CD8 T cells also uniformly expressed PD-L1, even more so than microglia, neutrophils, and some monocytes. Interestingly, among CD8 T cells, 8.8 TCR^–^ cells had lower MFI for PD-1 and lower frequency of PD-1^+^ cells than 8.8 TCR^+^ cells (Figure 5C and Supplemental Figure 3A). Both 8.8 TCR^+^ and 8.8 TCR^–^ cells had similar MFI and frequency of PD-L1 expression (Supplemental Figure 3, B and C). Lastly, due to the increased frequency of MoDCs and MFs in the spinal cord versus the brain, there was increased MFI for PD-L1 among CD45^+^ cells in the spinal cord (Supplemental Figure 3D). To further characterize checkpoint inhibitor expression on CD8 T cells during disease, we evaluated PD-1, TIM-3, LAG3, TIGIT, and CTLA-4 expression in the spleen and CNS. Both 8.8 TCR^+^ and 8.8 TCR^–^ CD8 T cells expressed high levels of PD-1, TIM-3, LAG3, and TIGIT but only low levels of CTLA-4 in the CNS, whereas in the spleen only a small proportion of the cells expressed these molecules (Figure 5, D and E, and Supplemental Figure 3E). These data suggest that the disease is self-resolving due to signaling through immune checkpoint molecules. Furthermore, CD11c expression seems to be a characteristic of CD8 T cells with terminal effector function in the CNS during CD8-EAE.

### Inflammation during CD8-EAE is restrained by PD-1 signaling.

**Figure 6:**
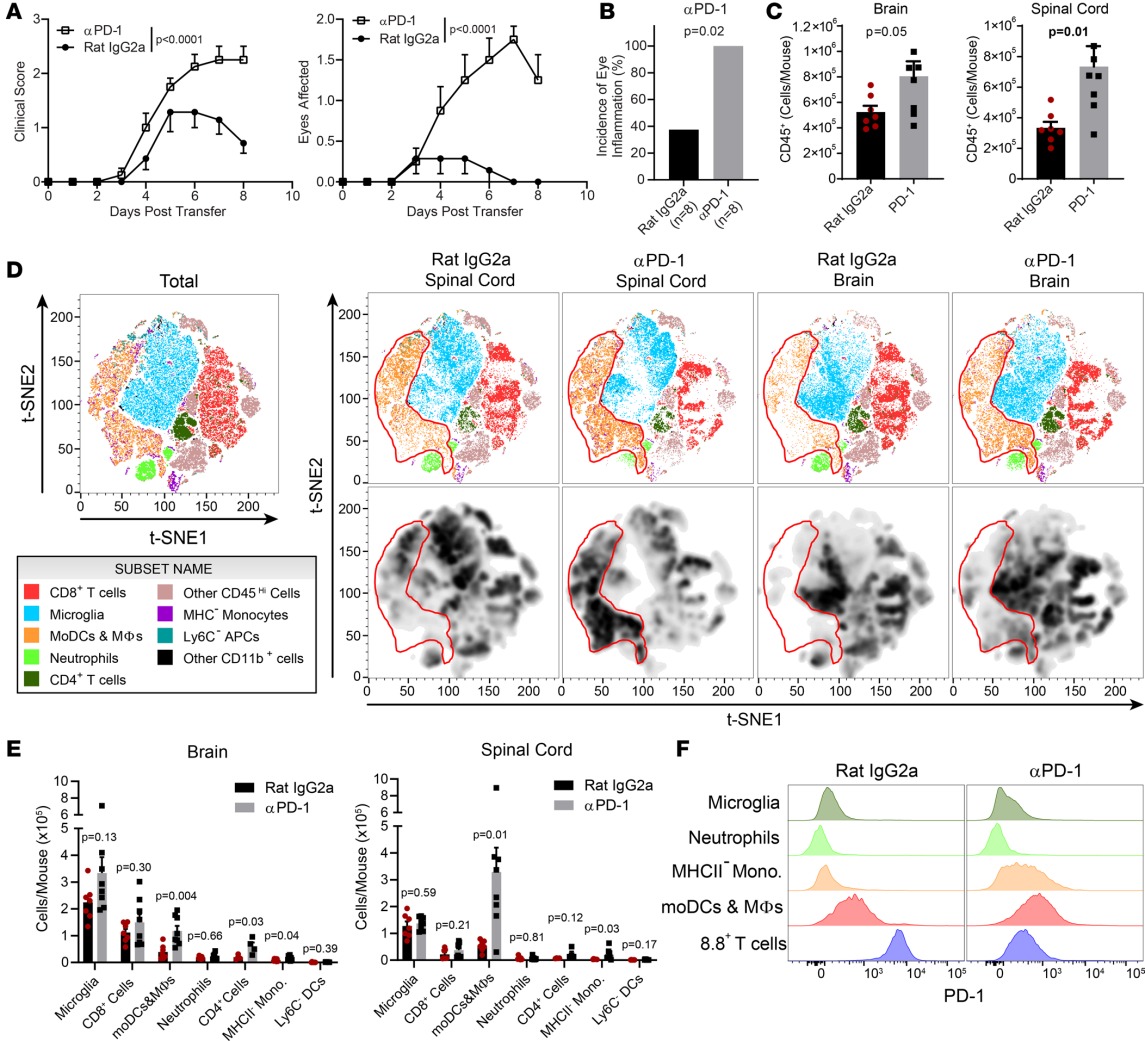
Blocking PD-1 signaling exacerbates CD8-EAE. Recipient mice received 8.8 T cells and were treated daily with 200 μg aPD-1 or isotype mAb (Rat IgG2a) starting from the day of CD8-EAE induction (*n* = 7–8 mice per group; compiled from 3 independent experiments). (**A**) Clinical scores and the number of inflamed eyes over time are shown. Statistical significance was determined by 2-way ANOVA. (**B**) Incidence of eye inflammation in mice treated with aPD-1 or isotype mAb. Significance was determined by the χ^2^ test. (**C**) Number of CD45^+^ cells from the brain and spinal cord of mice treated with aPD-1 and sacrificed on day 8 after CD8-EAE induction. (**D**) t-SNE clustering of CD45^+^ cells from multicolor flow cytometry data. CD8^+^ T cells were defined as CD8^+^CD3^+^; microglia as CD11b^+^Sall1^+^; MoDCs and macrophages as Sall1^–^CD11b^+^Ly6G^lo/–^Ly6C^+^MHCII^+^; neutrophils as Sall1^–^CD11b^+^Ly6G^hi^Ly6C^Int^; CD4 T cells as CD4^+^CD3^+^. Other CD45^hi^ cells did not express lineage markers stained in this panel. MHCII^–^ monocytes were Sall1^–^CD11b^+^Ly6G^lo/–^Ly6C^+^MHCII^–^; Ly6C^–^ APCs were Sall1^–^CD11b^+^Ly6C^–^MHCII^+^; other CD11b^+^ cells did not express Sall1, Ly6C, Ly6G, and MHCII. (**E**) Quantification of cell numbers based on gating from **D**. (**F**) Histograms depicting expression of PD-1 among cell populations. For **C**–**F**, statistical significance was determined by a 2-tailed unpaired *t* test.

The high expression of PD-1 and other immune checkpoint molecules on CD8 T cells in the CNS suggested that they curtail neuroinflammation in CD8-EAE. To test this hypothesis, we blocked PD-1 signaling during EAE using blocking mAbs against either PD-1 or PD-L1. Blocking PD-1 or PD-L1 exacerbated motor dysfunction and eye inflammation during CD8-EAE (Figure 6, A and B, and Supplemental Figure 4, A and B). Blocking PD-1 increased the number of immune cells in the brain and, to a greater extent, in the spinal cord (Figure 6C). Characterization of immune cells showed that the increase in cell numbers was primarily caused by an increase in inflammatory monocyte-derived cells (Figure 6, D and E). Anti–PD-1 treatment reduced the MFI of PD-1 on CD8 T cells in the CNS (Figure 6F and Supplemental Figure 4C) but had only moderate effects on PD-L1 expression on CD45^+^ cells in the CNS (Supplemental Figure 4, D and E). We cannot formally rule out the possibility that the injected anti–PD-1 mAb blocked the binding of the mAb used to ex vivo stain PD-1 on cells for flow cytometric analysis. However, we observed increased staining for PD-1 on monocytes, monocyte-derived dendritic cells, and macrophages in the samples from anti–PD-1 mAb–treated mice, while in the same samples, staining for PD-1 on 8.8 T cells was reduced (Figure 6F), indicating no significant competition between the blocking and staining mAbs. Lastly, we found a modest increase in the proportion of 8.8 TCR^–^ CD8 T cells of anti–PD-1–treated mice (Supplemental Figure 4F). Together, these data indicate that disease severity in this EAE model is limited by PD-1 signaling.

Over the course of our investigation, we noted that some aged 8.8 mice develop spontaneous overt signs of EAE (sEAE), which agrees with a previous report (13). Although animals that developed sEAE were rare, we have included in vitro analyses of a mouse with sEAE. We activated splenocytes from this mouse in vitro and compared their phenotype to that of cells from healthy-looking mice. Cells from the sEAE mouse exhibited a more activated phenotype, characterized by lower PD-1 expression and increased IL-2 and TNF expression. TIM-3 and IFN-γ expression was similar between the sEAE mouse and the normal mouse (Supplemental Figure 4G). Although these observations need to be validated by comparing cells from larger numbers of mice, our data thus far suggest that the immune status of naive donor mice is not uniform and may contribute to variability in CD8-EAE.

*Rapamycin-treated 8.8 CD8 T cells have enhanced encephalitogenicity*. Our data on the phenotype of CD8 T cells and the blockade of PD-1/PD-L1 suggest that the monophasic disease course in our CD8-EAE model results from the elimination/anergy of CD8 T cells by checkpoint inhibitor molecules, leading to the resolution of CNS inflammation. It is known that memory T cells exhibit resistance to such inhibitory mechanisms (17). We therefore tested whether induction of memory-like cells could exacerbate or extend disease in our CD8-EAE model by treating 8.8 CD8 T cells during in vitro activation with rapamycin or 2-deoxyglucose (2-DG), which have been shown to induce a memory phenotype in CD8 T cells with enhanced and prolonged effector function in vivo (18, 19).

**Figure 7:**
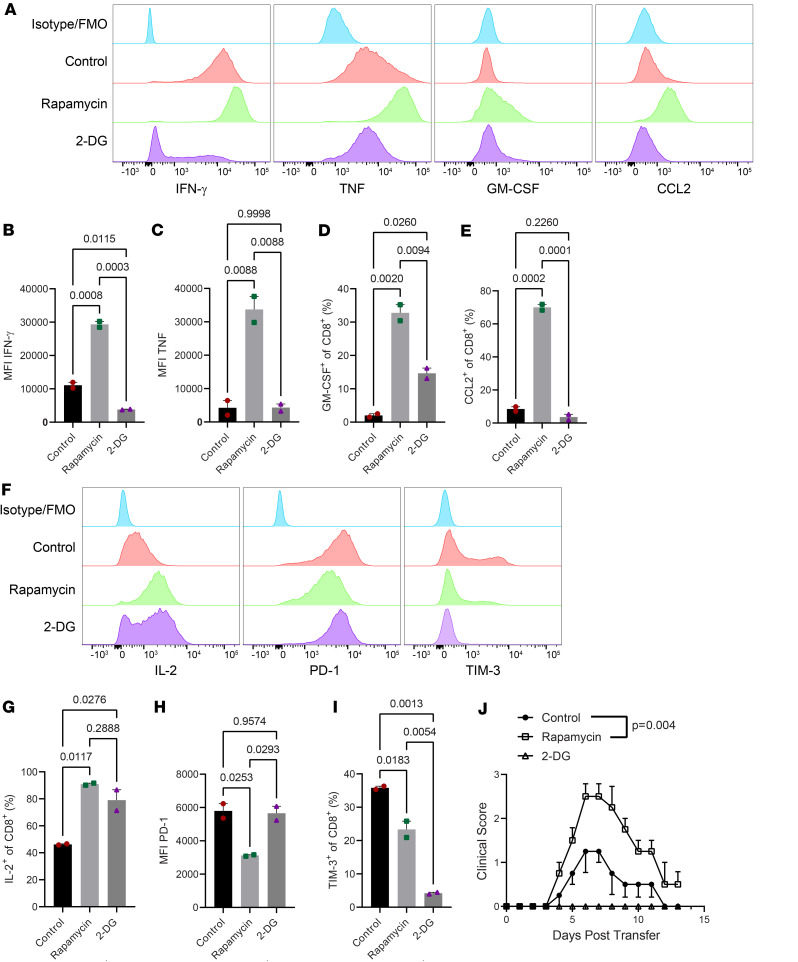
Rapamycin-treated 8.8 CD8 T cells induce more severe disease. The 8.8 CD8 T cells were activated in Tc1 conditions with 50 nM rapamycin (Rapa) or 4 mM 2-deoxyglucose (2-DG). (**A**) Expression of IFN-γ, TNF, GM-CSF, and CCL2 in CD8^+^ cells after 3-day stimulation with MBP_79-87_. (**B** and **C**) MFI of IFN-γ and TNF among IFN-γ^+^ and TNF^+^ cells. (**D** and **E**) Frequency of GM-CSF^+^ and CCL2^+^ cells among CD8^+^ cells. (**F**) Expression of IL-2, TIM-3, and PD-1 by CD8^+^ cells. (**G**) MFI for PD-1 on CD8^+^ cells. (**H**) Frequency of IL-2^+^ cells among CD8^+^ cells. (**I**) Frequency of TIM-3^+^ cells among CD8^+^ cells. Significance for **B**–**E** and **G**–**I** was determined by 1-way ANOVA with multiple comparisons testing. (**J**) Following first activation, cells were rested/expanded for 3 days in IL-2 media supplemented with 50 nM rapamycin or 1 mM 2-DG. In total, 2 × 10^7^ CD8 T cells were then transferred to recipient mice to induce CD8-EAE. Clinical course is shown; *n* = 4 for control and rapamycin; *n* = 2 for 2-DG. The significance of **J** was determined using a 2-way repeated measures ANOVA.

Rapamycin and 2-DG treatments differed in their ability to modify the phenotype of 8.8 CD8 T cells. Rapamycin strongly enhanced the production of inflammatory cytokines and chemokines, including IFN-γ, TNF, GM-CSF, and CCL2, whereas 2-DG only enhanced GM-CSF production (Figure 7, A–E). The enhanced production of IL-2 by CD8 T cells is associated with a memory phenotype and improved survival (20). Both rapamycin and 2-DG treatments increased the frequency of IL-2–expressing CD8 T cells during their culturing (Figure 7, F and G). The treatments also affected the expression of PD-1 and TIM-3, with rapamycin reducing the expression of both PD-1 and TIM-3, whereas 2-DG did not affect PD-1 expression but markedly reduced the frequency of TIM-3–expressing CD8 T cells (Figure 7, H and I). Treatment with 2-DG, but not rapamycin, upregulated TCF-1, a marker of stem cell memory T cells (Supplemental Figure 5A). We then transferred 2-DG– and rapamycin-treated 8.8 CD8 T cells to recipient mice to induce CD8-EAE. Rapamycin-treated cells induced exacerbated disease compared with control Tc1 cells, whereas 2-DG–treated cells were not encephalitogenic (Figure 7J and Supplemental Figure 5B). Although manipulation of CD8 T cell phenotype in the context of CD8-EAE requires further exploration, our data nonetheless suggest that inducing a memory-like phenotype could enhance pathogenicity of CD8 T cells.

### CD8-EAE pathology is dependent on IFN-γ.

**Figure 8:**
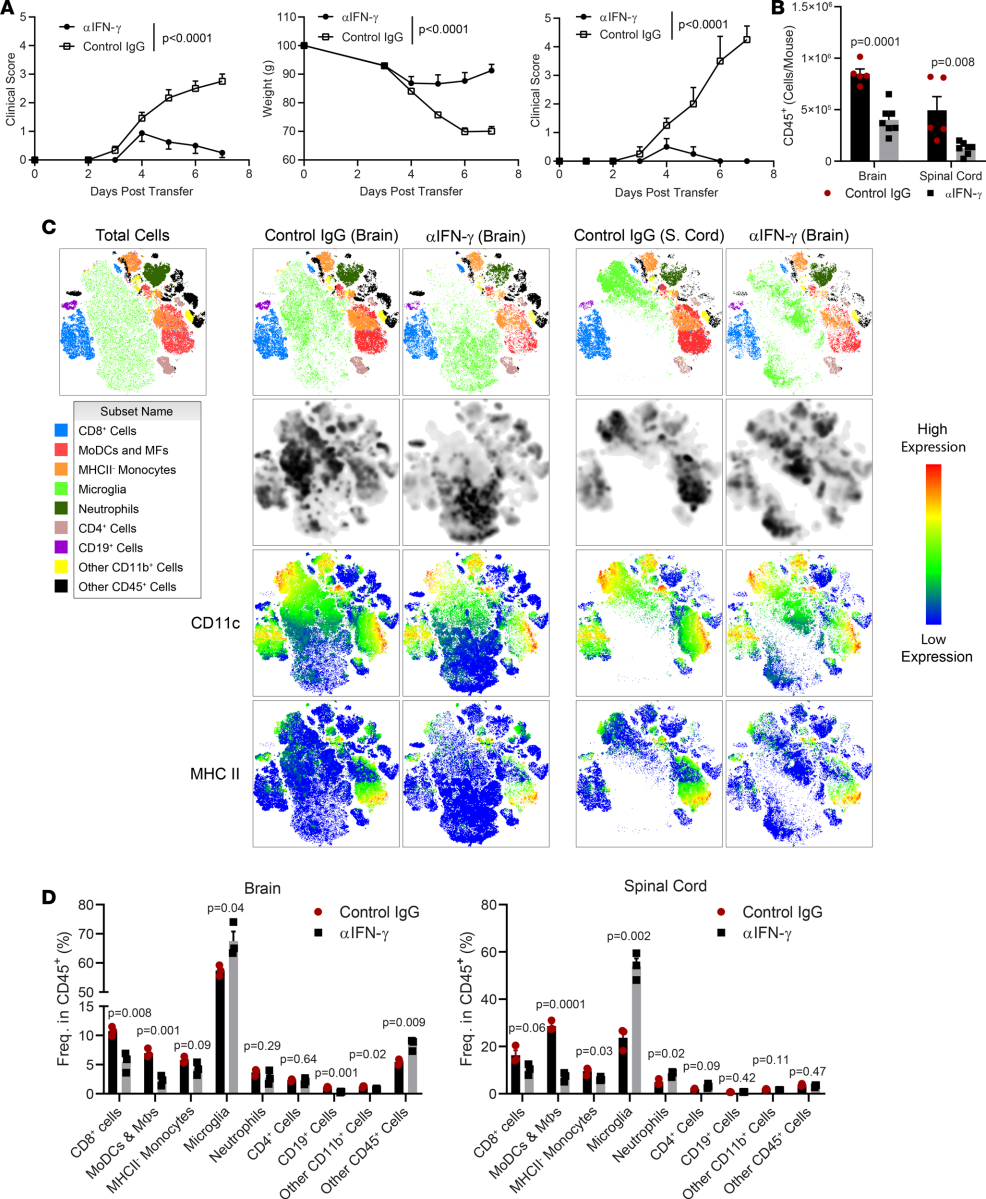
Blocking IFN-γ suppresses CD8-EAE. CD8-EAE was induced by transfer of 2 × 10^7^ CD8 T cells cultured in Tc1 conditions for 3 days, before being rested/expanded for 3 days before transfer. (**A**) Clinical scores and change in weight for CD8-EAE mice treated with 200 mg/day anti–IFN-γ mAb or control IgG; *n* = 8 per group, with data compiled from 2 experiments. The right panel shows clinical scores from a second independent experiment in which mice developed fulminant disease and were treated with either anti–IFN-γ or control IgG. *n* = 4 per group. Significance was determined by 2-way ANOVA. (**B**) Numbers of CD45^+^ cells isolated from the brain and spinal cord on day 7 after transfer; *n* = 5–8 per group compiled from 2 experiments. (**C**) t*-*SNE clustering of CD45^+^ cells isolated from the brain and spinal cord. CD8^+^ T cells were defined as CD45^+^CD8^+^; MoDCs and MFs as D45^hi^CD11b^+^Ly6G^lo/–^Ly6C^+^MHCII^+^, neutrophils as CD45^hi^CD11b^+^Ly6G^hi^Ly6C^Int^; monocytes as CD45^hi^CD11b^+^Ly6G^lo/–^Ly6C^+^MHCII^–^; microglia as CD45^lo^CD11b^+^; and CD4 T cells as CD45^hi^CD4^+^. B cells were defined as CD45^+^CD19^+^CD4^–^CD8^–^. Other CD11b^+^ cells were defined as CD45^+^CD11b^+^ cells that did not express other markers. Other CD45^+^ cells did not express other lineage markers stained in this panel. (**D**) Quantification of the frequency of cell populations from **C** among CD45^+^ cells in the brain and spinal cord.

We next sought to determine which mechanisms control pathology in CD8-EAE. Neutralizing GM-CSF with mAb moderately suppressed disease (Supplemental Figure 6A), indicating a minor contribution of GM-CSF to pathology in CD8-EAE. This finding aligns with the report that GM-CSF is dispensable in CD4-EAE in C3HeB/FeJ mice (21), which contrasts with the findings that GM-CSF is essential for CD4-EAE in other mouse strains tested (21, 22). It is possible that GM-CSF plays a lesser role in CD8-EAE than in CD4-EAE, regardless of mouse strain, because the encephalogenicity of CD8 T cells relies on mechanisms distinct from those of CD4 T cells. In earlier studies, intrathecal transfer of MBP-specific CD8 T cells caused neuroinflammatory disease that could be blocked by anti–IFN-γ mAb (10). We tested whether IFN-γ is also important in our CD8-EAE model and found that disease was potently suppressed by i.p. injections of anti–IFN-γ mAb. Anti–IFN-γ mAb even blocked fulminant disease in experiments in which nearly all control animals quickly succumbed to CD8-EAE (Figure 8A). In agreement with clinical disease suppression, mice treated with anti–IFN-γ mAb had reduced numbers of CD45^+^ cells in both the brain and spinal cord (Figure 8B). We then sought to characterize the effects that blocking IFN-γ had on CNS inflammation. Clustering of CD45^+^ cells from the brain and spinal cords revealed reduced frequencies of MoDCs and MFs in both the brain and spinal cord (Figure 8, C and D). CD11c-expressing microglia in both the brain and spinal cord were also reduced (Supplemental Figure 6B), suggesting diminished microglia activation in anti–IFN-γ mAb–treated CD8-EAE mice. CD8 T cells were also reduced in frequency in the brain, but not in the spinal cord (Figure 8D). CD8 T cells from anti–IFN-γ mAb–treated animals had reduced expression of PD-1, indicating reduced activation (Supplemental Figure 6C). Expression of PD-L1 among both CD11b^+^ cells and CD45^+^ cells was reduced, owing primarily to fewer moDCs and MFs in the CNS (Supplemental Figure 6, D–F). These data agree with the reports that IFN-γ is essential in CD8-EAE (23, 24), which is in stark contrast with its suppressive role in CD4-EAE (25, 26). Furthermore, these data indicate that IFN-γ promotes monocyte infiltration into the CNS and that monocytes are a major effector cell type in CD8-EAE.

### CNS infiltration by CD8 T cells is concomitant with local innate immune activation.

**Figure 9:**
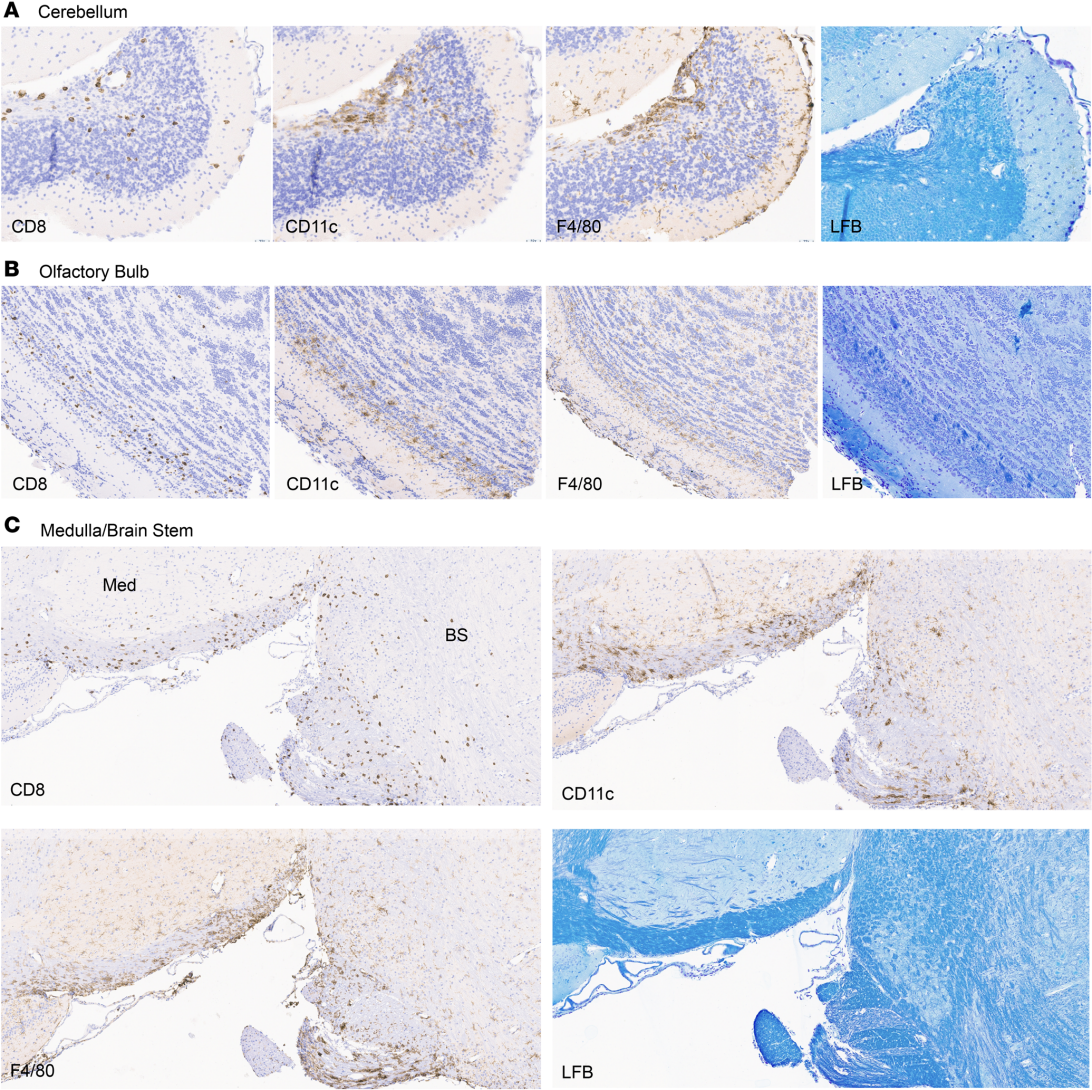
CD8 T cell infiltration in CD8-EAE is concomitant with regional innate immune activation. Sagittal sections of the brain were stained with Luxol fast blue for myelin, and IHC was performed for CD8, CD11c, and F4/80. (**A**–**C**) Inflammatory foci in the cerebellum (**A**), olfactory bulb (**B**), and medulla/brain stem (**C**) are shown. Original magnification, ×200. CD8, CD11c, and F4/80 are markers that were stained in immunohistochemistry. LFB, luxol fast blue; med, medulla; BS, brain stem.

**Figure 10:**
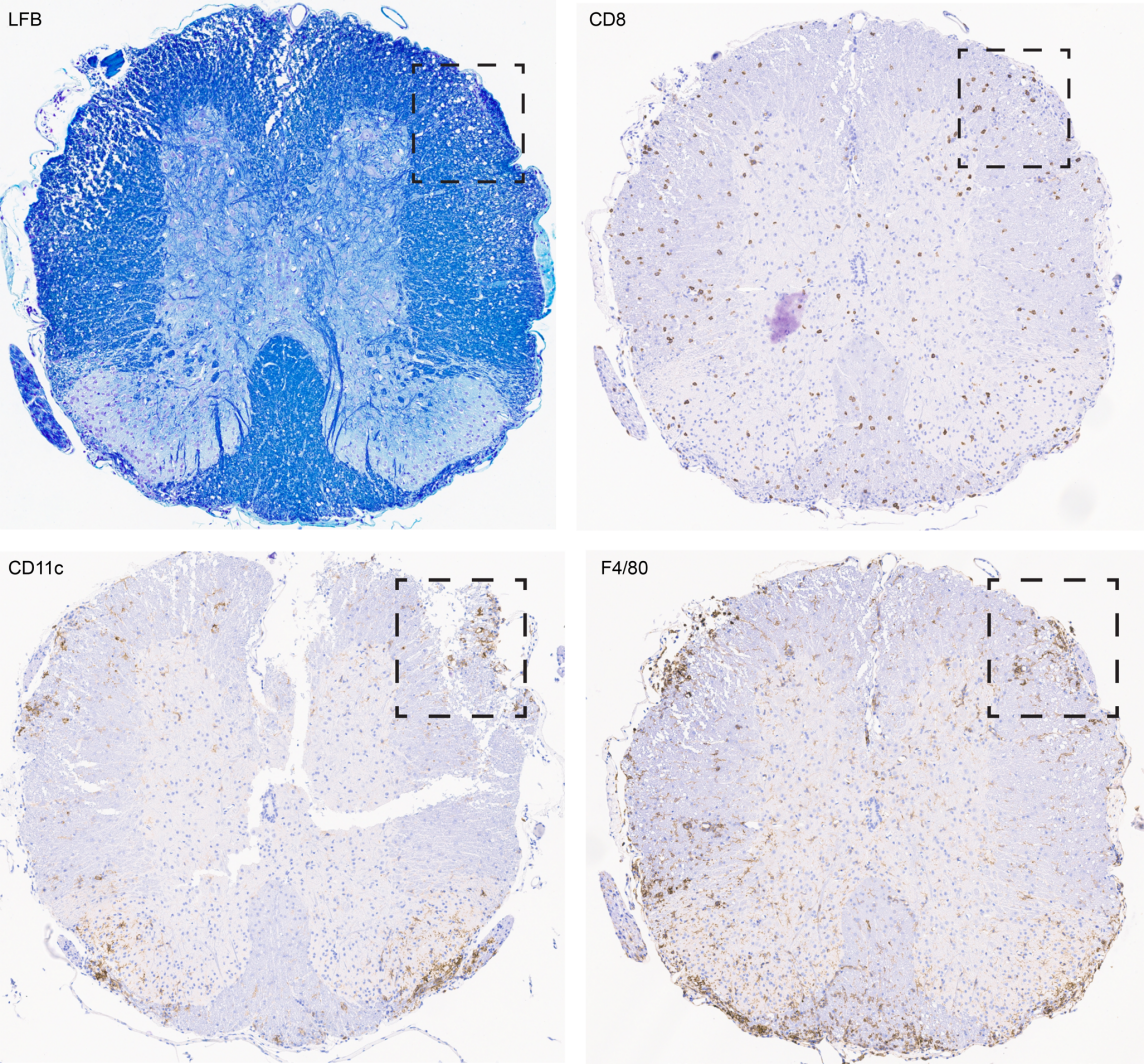
CD8 T cells infiltrate into the white and gray matter of the spinal cord during CD8-EAE. Sagittal sections of the spinal cord were stained with Luxol fast blue for myelin, and IHC was performed for CD8, CD11c, and F4/80. An example of inflammatory foci is marked by a box. Original magnification, ×200.

To further characterize the CNS pathology caused by adoptive transfer of 8.8 T cells, we performed histological analyses of the brains and spinal cords of mice with CD8-EAE. IHC staining revealed CD8 T cell infiltration in both the brain and spinal cord (Figure 9 and Figure 10). A widespread, diffuse CD8 T cell infiltration was observed, concomitant with regional activation of innate immune cells. CD8 T cell infiltration was observed across multiple brain regions, with the highest density in and around the brain stem, cerebellum, and olfactory bulb (Figure 9, A–C). In agreement with our flow cytometry analysis, the presence of CD8^+^ cells was accompanied by macrophage/microglia activation as assessed by IHC for CD11c and F4/80 (Figure 9). In contrast to CD4-EAE (27), CD8 T cells could be found in both the gray and white matter of the spinal cord (Figure 10). Inflammatory foci, however, were primarily restricted to the white matter. Notably, in both the brain and spinal cord, demyelination was mild and infrequent, despite considerable innate immune activation, which may be due to the short duration of the disease or the absence of other factors required for demyelination that are present in CD4-EAE. Nevertheless, inflammatory foci were observed in both the brain and the spinal cord in myelinated regions of tissue, consistent with the MBP specificity of the transferred 8.8 T cells. These data agree with our flow cytometry analysis, which shows that CD8 T cells infiltrate both the brain and the spinal cord, resulting in regional innate immune activation and inflammation.

## Discussion

We have developed an adoptive CD8-EAE model that relies on the transfer of CD8 T cells that express a transgenic TCR specific for MBP. The model is simple, requiring a single activation of CD8 T cells in vitro before their i.v. transfer into naive WT recipient mice, without the need for pertussis toxin injections. This is an uncommonly minimalist approach for a CD8-EAE model, as those described in the literature typically require additional manipulations to induce CNS autoimmunity, such as irradiation of recipient mice, transgenic recipient mice that express pseudoautoantigens, direct injection of CD8 T cells into the CNS, or viral infection. The drawback of these more complex models is not only the added technical difficulty but also that they are further removed from physiological processes/mechanisms, possibly representing them less realistically than our model. Furthermore, our model is robust, as mice reliably develop clinical disease only 4 days after receiving CD8 T cells, with disease peaking 7 days after T cell transfer and mice fully recovering 2 weeks after disease induction. In our experience, after optimizing multiple parameters, we have developed a simple protocol that reproducibly induces disease of moderate to high severity, without the excessive mortality often seen in our early experiments. As in other CD8-EAE models, mice exhibit varied outward signs of disease, which can make scoring disease severity difficult. However, a measurable parameter objectively reflecting disease severity was weight loss. Notably, we observed a strong correlation between the clinical score and weight loss, validating the scoring scale as a credible measure of disease severity.

Regarding the usefulness of our CD8-EAE model, we believe its simplicity, reproducibility, and physiological nature make it a valuable tool for studying the mechanisms of CD8 T cell–mediated neuroinflammation, as demonstrated by some of our experiments shown here (e.g., the role of IFN-γ). From the perspective of studying potential therapies targeting CD8 T cell–mediated pathogenic processes, the self-resolving disease course could limit therapeutic studies to a semiprophylactic treatment regimen, serving as a predictor for therapeutic effects when treatments are initiated during the ongoing disease. This limitation could hopefully be overcome by devising an approach to extend disease, as we attempted by treating 8.8 CD8 T cells with rapamycin and 2-DG. Still, our CD8-EAE model could be combined with a CD4-EAE model to generate a hybrid CD8/CD4-EAE model that may better represent MS than either CD8- or CD4-EAE models alone. A hybrid EAE model has been explored, utilizing the transfer of naive 8.8 CD8 T cells into mice with CD4 T cell–initiated EAE (14). In this scenario, the development of an autoreactive CD8 T cell response is secondary to CD4 T cell–induced neuroinflammation, with fewer CD8 T cells in the CNS than CD4 T cells. However, such a delay in the CD8 T cell response might not occur in MS, and cotransfer of activated 8.8 CD8 T cells with encephalitogenic CD4 T cells could more closely reflect MS pathogenesis. Owing to persistent neuroinflammation driven by CD4 T cells, such a hybrid EAE model would likely not be monophasic, making it useful for testing therapies in the context of CNS autoimmunity driven by both CD4 and CD8 T cells, which is likely the case in MS.

Experimental findings suggest that myelin-specific CD8 T cells direct CNS inflammation toward the brain more than CD4 T cells do, as transfer of autoreactive CD8 T cells into CD4-EAE enhanced brain inflammation, resulting in an increased incidence of atypical EAE (14). The mechanism by which CD8 T cells cause greater brain inflammation than CD4 T cells is unknown, but it may involve differential expression of adhesion molecules. Melanoma cell adhesion molecule (MCAM) is expressed by a subset of human effector CD8 T cells, and blocking its activity reduces transmigration in a human BBB model (28). Our data support the concept that subsets of CD8 T cells express adhesion molecules that could bias their accumulation toward the brain. In the CNS of CD8-EAE mice, ~80% of CD8 T cells expressed CD11c, far exceeding their systemic frequency (~5) (29) and their frequency prior to transfer (~15%) into recipient mice. CD11c^+^ CD8 T cells have not been described previously in the context of CNS autoimmunity, neither in EAE nor in MS. It would be highly relevant to determine whether CD8 T cells in some other CD8-EAE models — and, particularly, in MS lesions — are CD11c^+^. Currently, it is unclear whether CD11c upregulation on CD8 T cells is a peculiarity of our model or a common phenomenon in the inflamed CNS. It is also unknown whether and how CD11c is important for CD8 T cell function in CNS autoimmunity. CD11c, also known as α_X_integrin, pairs with b_2_ integrin to form complement receptor 4 (CR4). In addition to myeloid cells, CR4 can be expressed on lymphocytes, including T cells (30, 31). KO of CD11c (and therefore CR4) on T cells suppressed adoptive CD4-EAE (32). Thus, it seems possible that CD11c directly contributes to the pathogenicity of autoreactive CD8 T cells in EAE. However, it is unlikely that CD11c^+^ and CD11c^–^ CD8 T cells only differ in the expression of this molecule. In viral infections, CD11c^+^ CD8 T cells exhibit greater inflammatory effector function than their CD11c^–^ counterparts (33). The effector functions of CD11c^+^CD8^+^ T cells appear to be centered on IFN-γ production, rather than on their cytolytic function (29), which is in agreement with our data showing that CD8-EAE is IFN-γ dependent. Characterization of phenotypic and functional differences between CD11c^+^ and CD11c^–^ CD8 T cells is relevant not only in the context of CNS autoimmunity, given that little is known about this topic in general. It appears that our CD8-EAE model could be useful in such studies.

In CD4-EAE, both Th1 and Th17 cells are sufficiently pathogenic to induce EAE (34). Th1 and Th17 cells are present in MS lesions, which, together with observations from EAE, indicate that both Th subsets are pathogenic in MS. Similarly, Tc1 and Tc17 cells can be found in MS lesions (7), but it is unknown whether these CD8 T cell subsets differ in their pathogenicity because the encephalitogenicity of Tc17 cells has not been directly tested. In an atypical model of active EAE, Tc17 cells themselves did not induce EAE but provided crucial help in the differentiation of encephalitogenic Th17 cells, which then caused EAE (35). It is unclear if, and to what extent, this indirect proencephalitogenic function of Tc17 cells is relevant in other EAE models. In our testing of Tc1 versus Tc17 cells, we were surprised to find that Tc1 cells were notably more encephalitogenic than Tc17 cells. Although we are not certain why this is the case, a plausible explanation is that the greater IFN-γ production by Tc1 cells renders them more pathogenic. Indeed, we found that IFN-γ was an essential pathogenic mediator in CD8-EAE, as neutralization of IFN-γ abrogated even fulminant disease. This finding aligns with other studies demonstrating that CD8-EAE development is dependent on IFN-γ (10, 23, 24). However, the ability for 8.8 CD8 T cells to promote brain inflammation in a model of CD4-EAE was independent of IFN-γ (14). This is opposite to the protective role of IFN-γ in CD4-EAE, with IFN-γ–KO mice developing exacerbated disease (26). In contrast to CD4-EAE, IFN-γ appears to be proinflammatory in MS, as administration of IFN-γ to patients with MS during a clinical trial in the 1980s caused an increase in MS relapse rate (36). Thus, the role of IFN-γ in MS is likely better recapitulated in CD8-EAE than in CD4-EAE, suggesting that IFN-γ production of CD8 T cells is a relevant pathogenic mechanism in MS.

It is not known whether Tc cell cytotoxicity contributes to their encephalitogenicity; however, if it is important, the low encephalitogenicity of Tc17 cells could also be explained by a lack of cytotoxicity. We and others have shown that Tc17 cells do not express granzyme B or perforin and are not cytotoxic (35, 37, 38), although perforin-independent (38) and FasL-dependent (39) cytotoxicity of Tc17 cells has been described. Perforin produced by CD8 T cells is important in disrupting the BBB and in the development of brain inflammation (40). Pharmacological inhibition of perforin largely reversed the neurological deficit caused by autoreactive CD8 T cells that disrupted the BBB (41). The 8.8 T cells activated by vaccinia infection needed to be perforin (and IFN-γ) sufficient to induce neuroinflammation (13), strongly suggesting that perforin is an obligatory factor for encephalogenicity of CD8 T cells. Information on the role of granzyme B in EAE, particularly in CD8-EAE, is scarce. It has been demonstrated that inhibiting granzyme B in CD4-EAE reduces axonal and neuronal injury, while maintaining myelin integrity, but it does not decrease infiltration of CD4 and CD8 T cells into the CNS (42). CD8 T cells expressing granzyme B were observed in proximity to or attached to oligodendrocytes or demyelinated axons, indicating that granzyme B contributes to their pathogenic function (43). A recent study has shown that patients with MS with progressive disease course have greater numbers of CD8 T cells with granzyme B expression than patients with relapsing remitting MS, and their expression positively correlated with disability and progression of MS (44). T-bet was required for the expression of granzyme B in these CD8 T cells (44). Given that granzyme B and perforin are part of the same pathway, and perforin is required for the encephalogenicity of CD8 T cells, it implies that granzyme B is also required. Overall, direct evidence for the role of cytotoxic molecules in the encephalitogenicity of CD8 T cells is incomplete; however, it seems possible that they may be essential for EAE to develop when encephalitogenic CD4 T cells are absent, and CD8 T cells alone need to initiate and drive CNS inflammation. Even though Th17 cells are highly encephalitogenic, their function depends on T-bet upregulation and a shift in their phenotype toward Th1 (45). Tc17 cells do not express T-bet, but they can upregulate T-bet expression and switch to a Tc1-like, ex-Tc17 phenotype (37), analogous to ex-Th17 cells. We have also seen in our experiments with 8.8 Tc17 cells that, after second stimulation, a portion of them coexpresses IL-17 and IFN-γ, which signifies an ongoing Tc17 to Tc1 switch. It is likely that this switch will continue or even accelerate after the transfer of Tc17 cells into recipient mice. However, unlike Th17 cells, this switch does not seem to confer encephalitogenicity to Tc17/ex-Tc17 cells. Our findings suggest that further investigation into the encephalitogenicity of myelin-specific Tc17 cells is necessary to address the questions raised by our data.

It is unknown how IFN-γ facilitates CD8-EAE, but our data indicate that IFN-γ participates in monocyte activation and recruitment into the CNS, as blocking IFN-γ greatly reduced numbers of monocytic cells in both brain and spinal cord. Moreover, 8.8 CD8 T cells treated with rapamycin showed augmented IFN-γ production and caused severe disease, suggesting that rapamycin enhances the encephalitogenicity of 8.8 CD8 T cells by increasing their IFN-γ production. Indeed, rapamycin appeared to enhance the effector phenotype of 8.8 CD8 T cells overall, with increased IL-2 production and diminished checkpoint molecule expression. One potentially relevant mechanism is IFN-γ–dependent production of CCL2, which can be produced both by nonimmune cells, including astrocytes (46–49), and immune cells, including monocytes and microglia (50, 51). In agreement with this hypothesis, we found widespread CCL2 production by CNS immune cells in CD8-EAE, including monocytes, microglia, and CD8 T cells. Since blocking IFN-γ reduced the number of inflammatory monocytes, which are a source of CCL2, this may have led to reduced monocyte recruitment to the CNS.

Although our CD8-EAE model recapitulates some aspects of MS, its monophasic nature is a notable difference. Monophasic disease correlated with a high degree of PD-1 expression on CD8 T cells both in vivo and in vitro, and with high PD-L1 expression on most immune cells in the CNS, indicating that PD-1 signaling contributes to the self-resolving disease course. Accordingly, blockade of PD-1 or PD-L1 exacerbated ataxia in CD8-EAE. It has been shown that PD-1 signaling also limits CD4-EAE severity, as mice deficient for PD-1 or PD-L1 (but not PD-L2) develop more severe disease (52). These data suggest that strong signaling via immune checkpoint inhibitors dampens effector functions of 8.8 T cells, resulting in a monophasic disease course. Indeed, there is precedent for this concept, as TIM-3 KO exacerbated a vaccinia infection–induced model of CD8-EAE (53). The monophasic disease course in this model could be explained by the absence of Th responses, which are required for sustained CD8 T cell responses across various diseases and therapeutic settings (54). Our understanding of the role of CD8 T cells in MS may, therefore, benefit from comparison between CD8-EAE and hybrid CD4/CD8-EAE models. Given that our model initiates disease solely through CD8 T cells, this allows for the study of encephalitogenic mechanisms of these cells without interference from CD4 T cells. It should also be noted that the use of inbred experimental animals and monoclonal TCR transgenic mice to induce disease in our model is in stark contrast to the immunologic variance (55) and likely viral component (56) that characterize the development of MS in humans. MS likely involves the development of polyclonal T cell responses that may change over time (57), which is an element of the disease that remains to be captured by any MS model. Lastly, some mice with CD8-EAE developed apparent periocular inflammation. Notably, although uncommon, uveitis is associated with MS (58, 59) along with other ocular complications, such as the more well-known optic neuritis (60), which is often an early sign of MS. Whether CD8-EAE mice develop optic neuritis and the cause of the apparent uveitis remains to be determined.

Our histological analysis of the CNS from mice with CD8-EAE revealed interesting patterns of inflammation. In contrast to CD4-EAE, in which T cells localize to dense lesions in the white matter of the spinal cord, CD8 T cell infiltration was considerably more diffuse and widespread in both white and gray matter. While infiltration of CD8 T cells in both the brain and spinal cord correlated with macrophage/microglial activation, as seen in CD4-EAE, we did not observe prominent demyelination in CD8-EAE mice. This lack of demyelination in our model may be explained by the limited duration of the disease, which may be insufficient for demyelinated lesions to develop. Alternatively, CD8 T cell–mediated inflammation may lack a qualitative component (e.g., cytokine, chemokine, mediator) that mediates demyelination in CD4-EAE models. Given that mice in our CD8-EAE model fully recover from disease, it is unlikely that they develop significant, permanent axonal/neuronal damage/loss in their CNS. Reversible clinical deficits in EAE are primarily caused by transient inflammation-induced mitochondrial and axonal dysfunction, synaptic mechanisms, and BBB disruption, independent of demyelination (61). It would be interesting to directly compare the disease mechanisms between our CD8-EAE model and the monophasic CD4-EAE model, such as adoptive EAE in PL/J or B10/PL (both H-2^U^ haplotype) mouse strains (62).

In conclusion, we have established a convenient adoptive CD8-EAE model, characterized by inflammation in both the brain and spinal cord. The simplicity of the model, specifically the use of naive WT recipient mice, is unique among CD8-EAE models and is more likely to mimic physiological processes, potentially providing more relevant insights into processes in MS. The neurological deficits in our model differ from those in common CD4-EAE models and are perhaps more similar to MS. This is likely caused by more severe brain inflammation than in CD4-EAE models, although the specifics of inflammation driven only by CD8 T cells, including a self-resolving disease course, certainly have an effect on observable clinical deficits. We believe that our CD8-EAE model is a highly useful tool for studying an understudied aspect of CNS autoimmunity, despite its considerable relevance to MS. Therefore, using our model to investigate autoimmune neuroinflammation could lead to a better understanding of MS and open new avenues for its therapy.

## Methods

### Sex as a biological variable.

Both male and female mice were used in studies.

### Mice.

The 8.8 mice (on C3HeB/Fe/J background) were provided by Joan Goverman (University of Washington). WT C3HeB/FeJ mice were obtained from The Jackson Laboratory (stock no. 000658).

### Induction of CD8-EAE and treatments.

Splenocytes from 8.8 mice were isolated by mechanical dissociation followed by RBC lysis using RBC lysis buffer (BioLegend). Cells were plated at 2 × 10^6^ cells/mL in 6-well plates with 10 mL of IMDM + 2 mM L-glutamine and 10% heat-inactivated FBS. Cells were stimulated for 72 hours with 0.33 mg/mL MBP_79-87_ and recombinant cytokines, as indicated in the results. Cells were washed twice and then rested for 3 days in media containing 5 ng/mL IL-2 (PeproTech). Each day, cells were replated at 1 × 10^6^ cells/mL in fresh media supplemented with IL-2. For experiments where 2-DG and rapamycin were used, 8.8 T cells were stimulated for 3 days in Tc1 conditions supplemented with 50 nM rapamycin or 4 mM 2-DG. During rest, 2-DG–treated cells were treated with 1 mM 2-DG. In experiments in which cells were activated twice, cells were reactivated with anti-CD3 mAb (clone 145-2C11; Bio X Cell) and anti-CD28 mAb (clone 37.51; Bio X Cell). Plates were coated with anti-CD3 and anti-CD28 mAbs by covering the bottom of each well with 1 mg/mL anti-CD3 and 1 mg/mL anti-CD28 mAbs in PBS and incubating overnight at 37°C. Cultures were then restimulated for 2–3 days at 1 × 10^7^ cells/mL in 6-well plates with 10 mL media. CD8 T cells were harvested and purified by MACS separation by negative selection (Miltenyi Biotec) and resuspended in IMDM medium without FBS at 2 × 10^7^ cells/300 μL media that were supplemented with 0.2–0.4 μg IL-2 per mouse. In total, 2 × 10^7^ cells were transferred by i.v. tail injection into recipient mice. For blockade and detection of PD-1, the 29F.1A12 antibody or an isotype-matched mAb was used. For blockade and detection of PD-L1, the 10F.9G2 antibody or an isotype-matched antibody was used. For blockade and detection of GM-CSF, MP1-22E9 mAb was used.

### Clinical scoring.

Mice were scored using atypical EAE scale as follows: grade 1, hindlimb clasping and/or hyperactivity; grade 2, hindlimb weakness and/or spastic movement; grade 3, spastic movement and dragging of hind limbs when walking (typically presenting as severe loss of coordinated movement, not paralysis); grade 4, head tilt with body leaning, forelimb weakness; grade 5, moribund or death.

### Cell isolation and flow cytometry analysis.

To isolate the CNS mononuclear cells, blood was removed by cardiac perfusion with 60 mL PBS. The spinal cord was then flushed from the spinal column with PBS. The brains and spinal cords were combined and cut into small pieces, which were then incubated with Liberase TL (Roche) dissolved in RPMI at 0.7 mg/mL at 37°C for 30 minutes. The reaction was stopped by using complete media containing FBS, and the tissue was homogenized by pushing it through a 100 μm sterile filter with a syringe plunger. The homogenate was then centrifuged at 1500 RPM (300*g*) for 5 minutes and resuspended in 10 mL of 40% 1× Percoll-PBS (90% Percoll, 10% 10× PBS). It was then centrifuged at 800*g* without a brake at room temperature for 30 minutes. The pelleted cells were collected, diluted with PBS or media, and centrifuged at 1500 RPM (300*g*) for 5 minutes. The isolated cells were then stimulated with PMA (50 ng/mL; Sigma-Aldrich), ionomycin (500 ng/mL; Sigma-Aldrich), and 1 μL/mL GolgiPlug (BD Biosciences) for 4 hours at 37°C or stained without stimulation. After stimulation, the cells were washed with PBS containing 3% FBS (v/v). Cell surface antigens were then stained with mAbs in 100 μL of PBS/3% FBS for 20–30 minutes at 4°C. The cells were then washed and fixed with 100 μL of Fix and Perm Medium A (Thermo Fisher Scientific) for 20 minutes at room temperature, followed by another wash. The cells were permeabilized with Fix and Perm Medium B (Thermo Fisher Scientific) and then stained with mAbs against intracellular antigens in 100 μL of Fix and Perm Medium B and 100 μL of PBS/3% FBS for 1 hour or overnight. Finally, the cells were washed twice, resuspended in 500 μL PBS, and analyzed on a BD FACSAria Fusion flow cytometer (BD Biosciences).

### Histology and IHC.

Animals were perfused with 40 mL cold paraformaldehyde (PFA) solution. The spinal column and brain were then removed and placed in PFA overnight. The spinal cord was then dissected from the spinal column, cut into 4 pieces, before paraffin embedding. Hemispheres of the brain were separated before embedding. The spinal cord was sectioned into coronal sections, and the brain was cut into sagittal sections. Histological staining and immunolabeling were performed at the Children’s Hospital of Philadelphia Pathology Core Laboratory. F4/80 (Cell Signaling, 70076), Iba1 (Wako 019-19741), CD8a (Cell Signaling, 98941), and CD11c (Cell Signaling, 97585) mAbs were used to label formalin-fixed paraffin-embedded (FFPE) tissue slides. Immunostaining was performed on a Bond Rx automated staining system (Leica Biosystems). The Bond Refine polymer staining kit (Leica Biosystems DS9800) was used. The standard protocol was followed, except that the primary Ab incubation was extended to 1 hour at room temperature, and the postprimary step was omitted. Abs were diluted at 1:200, 1:2,000, 1:500, and 1:100, respectively. Antigen retrieval was performed with E2 (Leica Biosystems AR9640) retrieval solution for 20 minutes, apart from F4/80 where E1 (Leica Biosystems AR9961) was used. Slides were rinsed, dehydrated through a series of ascending ethanol and xylene concentrations, and then cover slipped. Kluver-Barrera staining was used on FFPE samples. Briefly, slides were deparaffinized and rehydrated to 95% ethanol. They were then immersed in 0.1% Luxol Fast Blue overnight at 56°C. Slides were then rinsed in 95% ethanol, followed by distilled water. After rinsing, the slides were quickly dipped into 0.05% lithium carbonate before differentiating in 70% ethanol. After differentiation, the slides were rinsed in deionized water before counterstaining in 0.1% Cresyl Etch Violet Solution for 10 minutes at 56°C. Slides were then differentiated in 95% ethanol, dehydrated to 100% ethanol, cleared with xylene, and coverslipped with cytoseal. Stained slides were then digitally scanned at 20× magnification on an Aperio AT2 slide scanner (Leica Biosystems). Histological evaluation was performed at the Comparative Pathology core at the University of Pennsylvania. Two sagittal sections of the brain and 4 coronal sections of the spinal cord were evaluated per animal.

### Statistics.

Incidence-based statistical tests were performed using the χ^2^ test. Statistical significance between groups for clinical courses were determined by 2-way ANOVA. Most other tests used a paired or unpaired 2-tailed *t* test, as appropriate. *P* values are listed.

### Study approval.

All animal studies were approved by Thomas Jefferson University IACUC.

### Data availability.

The underlying data used to generate the figures are available in the Supporting Data file or upon request.

## Author contributions

DH was responsible for designing research studies, conducting experiments, acquiring data, analyzing data, providing reagents, and writing the manuscript. LLWI, MS, SJ, AC, GA, and EK conducted the experiments. AR and GXZ provided reagents and expertise on research studies and edited the manuscript. BC was responsible for designing research studies, interpreting data, and writing the manuscript.

## Conflict of interest

The authors have declared that no conflict of interest exists.

## Funding support

This work was supported by the National Institutes of Health T32 training grant (T32AI134646, NIAID) and is subject to the NIH Public Access Policy. Through acceptance of this federal funding, the NIH has been given the right to make the work publicly available in PubMed Central.

## Supplementary Material

Supplemental data

Supplemental video 1

Supporting data values

## Version 1 05/22/2026

Electronic publication
