# Supplementary material for: CD11c+ CD8 T cells cause IFN-γ–dependent autoimmune neuroinflammation that is restrained by PD-1 signaling
Source: JCI Insight. 2026 May 22;11(10):e179789. doi: 10.1172/jci.insight.179789 (PMC13232716; doi:10.1172/jci.insight.179789)
Supplement: Supplemental data [file jciinsight-11-179789-s178.pdf]

SUPPLEMENTAL INFORMATION

Supplemental Figure 1

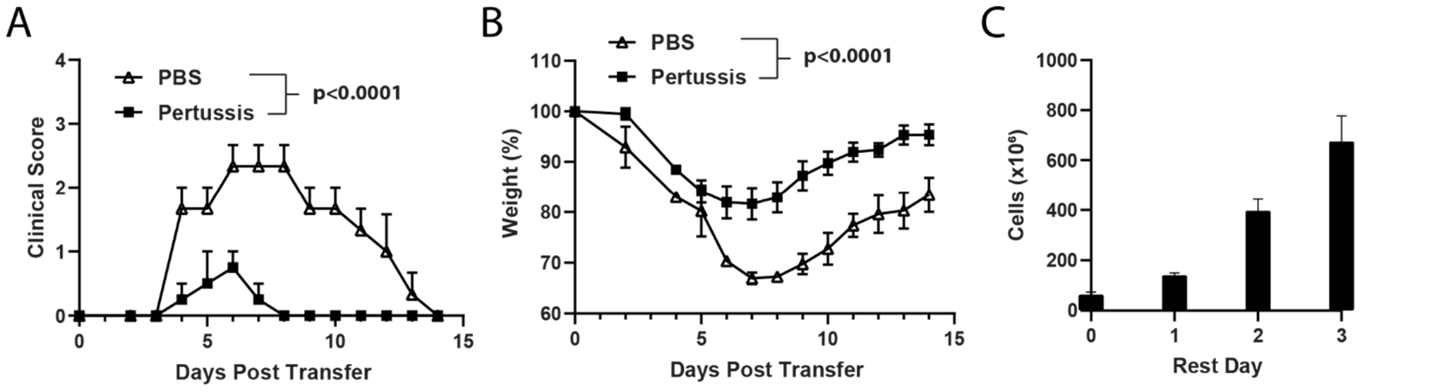

Supplemental Figure 1. A single activation is sufficient to render Tc1 cells encephalitogenic, and pertussis toxin suppresses Tc1-mediated EAE. Splenocytes from 8.8 mice were stimulated with the MBP_{79-87} peptide for 3 days under Tc1-polarizing conditions, which consisted of IL-12 (2 ng/mL) and IL-2 (5-10 ng/mL). After 72 h, cells were rested in 10 ng/mL IL-2; each day, cells were split and replated in fresh media supplemented with IL-2. On the third day, CD8 T cells were purified by MACS *via* negative selection, and 2 x 10^7 cells in 300 µL RPMI (media only without FBS) were i.v. injected into naïve C3HeB/FeJ recipients along with 0.4 µg IL-2 per mouse. **A)** Clinical course for CD8-EAE in mice that were injected i.p. with either 200 ng pertussis toxin or PBS on day 0 and 2 post transfer (n=3 mice per group). **B)** Change in weight during CD8-EAE. Significance was determined by Two-way ANOVA. **C)** Cell number in cultures over the course of rest/expansion following 3 days of activation. In the experiments shown, splenocytes from two 8.8 donor mice were used.

Supplemental Figure 2

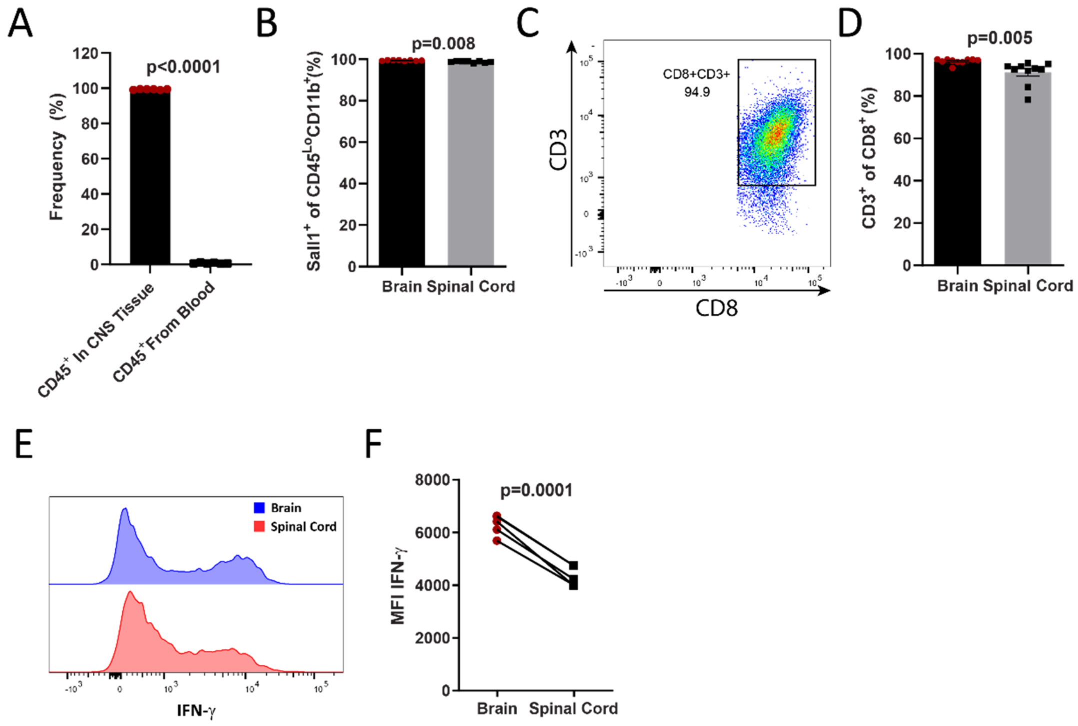

Supplemental Figure 2. Tc1 cells cause distinct inflammation in the brain and spinal cord. CD8-EAE was induced by i.v. transfer of 2 x 10^7 Tc1 cells. **A)** Quantification of cells from the CNS of mice with CD8-EAE that are resident/infiltrating *vs*. those deriving from blood. On day 7 post-transfer, to label $CD45^+$ cells in the blood, 10 µg of a fluorochrome-labeled anti-CD45 mAb was injected i.v. 5 min prior to perfusion and CNS isolation. After isolating cells from the CNS, the cells were stained with an anti-CD45 mAb conjugated to a different fluorochrome than the i.v.-injected anti-CD45 mAb, and the frequency of $CD45^+$ cells in CNS tissue and $CD45^+$ cells derived from blood was determined. **B)** Frequency of $Sall1^+$ cells among $CD45^{Lo}CD11b^+$ cells in the brain and spinal cord. **C)** Flow cytometry plot showing CD3 and CD8 expression among $CD45^+Sall1^-$ cells (non-microglial immune cells). **D)** Frequency of $CD3^+$ cells among $CD8^+$ cells in the brain and spinal cord. **E)** Histograms depicting IFN-γ expression among monocytes. **F)** MFI of IFN-γ^+ cells in $CD45^{Hi}CD11b^+Ly6G^{Lo/-}Ly6C^+$ monocytes from the brain and spinal cord. Significance of A-C was determined by an unpaired t-test. Significance for E was determined by a paired t-test.

Supplemental Figure 3

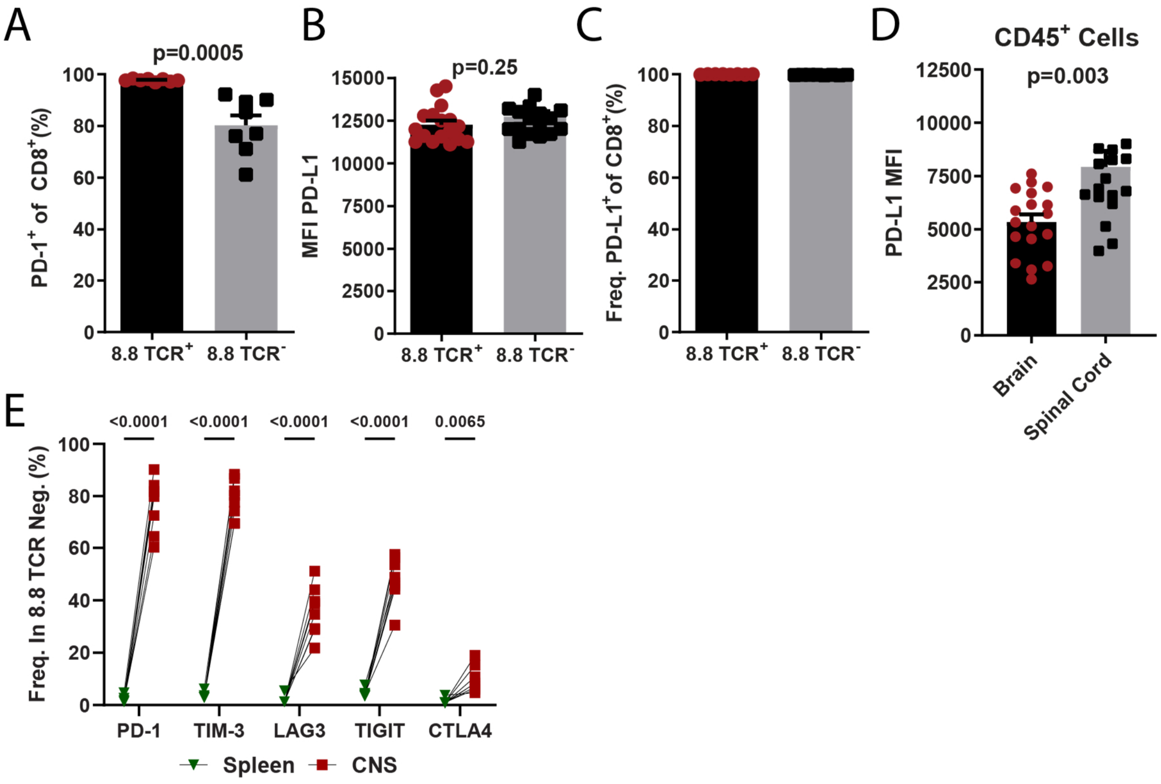

Supplemental Figure 3. CNS CD8 T cells in CD8-EAE are predominantly CD11c+ with a terminal effector phenotype. CD8-EAE was induced by i.v. transfer of 2 x 10^7 Tc1 cells, and mice were sacrificed 7 days after transfer. Brain and spinal cord cells were analyzed by flow cytometry. **A)** Frequency of PD-1+ cells among 8.8 TCR+ and 8.8 TCR- CD8 T cells from the CNS (brain + SC). **B)** MFI of PD-L1+ cells among CD8+ cells. **C)** Frequency of PD-L1+ cells among CD8+ cells. Significance for A-C determined by an unpaired t-test. **D)** MFI of PD-L1 among CD45+ cells. Significance was determined by a paired t-test. **E)** Frequency of cells expressing PD-1, TIM-3, LAG3, TIGIT, and CTLA4 among 8.8 TCR- CD8+ cells in the spleen and CNS. Significance was calculated by two-way ANOVA followed by Šídák's multiple comparisons test. N=8 mice.

Supplemental Figure 4

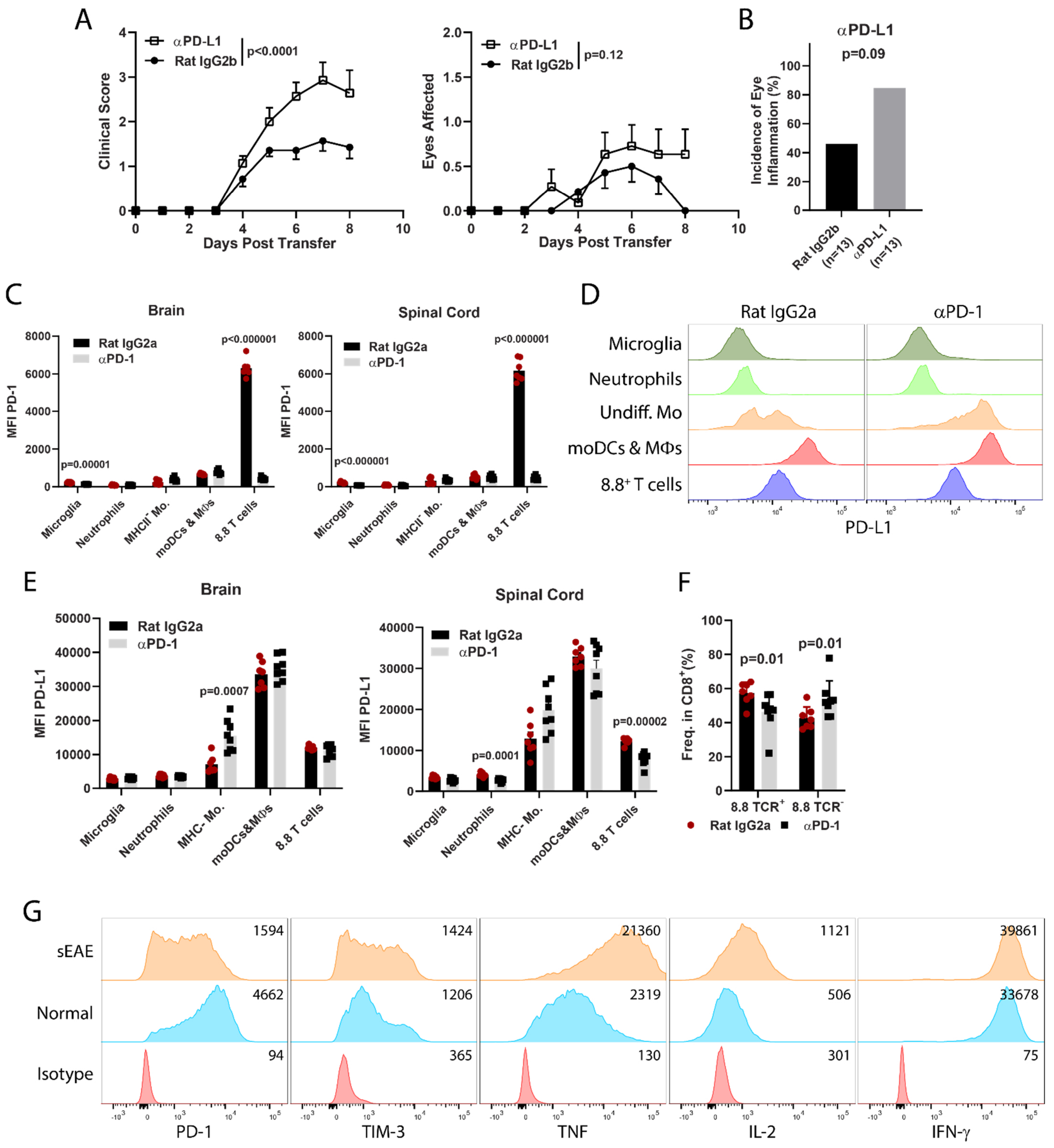

Supplemental Figure 4. Blocking PD-L1 exacerbates CD8-EAE. **A)** CD8-EAE was induced, and recipient mice were treated with 200 μg αPD-L1 or isotype mAb (Rat IgG2b) once per day starting from the day of CD8-EAE induction (n=14 per group; compiled from 3 independent experiments). Statistical significance was determined by two-way ANOVA. **B)** Incidence of eye inflammation in mice treated with αPD-L1, or isotype mAb. Significance was determined by the Chi-squared test. **C)** MFI for PD-1 in the brain and spinal cord among cell populations. **D)** Flow cytometry histograms depicting expression of PD-L1 among cell populations. **E)** MFI for

PD-L1 in the brain and spinal cord among populations. **F)** Frequency of 8.8 TCR^{+} and 8.8 TCR^{-} cells among CD8^{+} cells. For D-F, statistical significance was determined by a two-tailed unpaired t-test. **G)** Histograms depicting expression of PD-1, TIM-3, TNF, IL-2, and IFN-γ by 8.8 CD8 T cells stimulated for 3 days with MBP_{79-87} that were derived from an 8.8 mouse with spontaneous EAE (sEAE) or normal-appearing donors.

Supplemental Figure 5

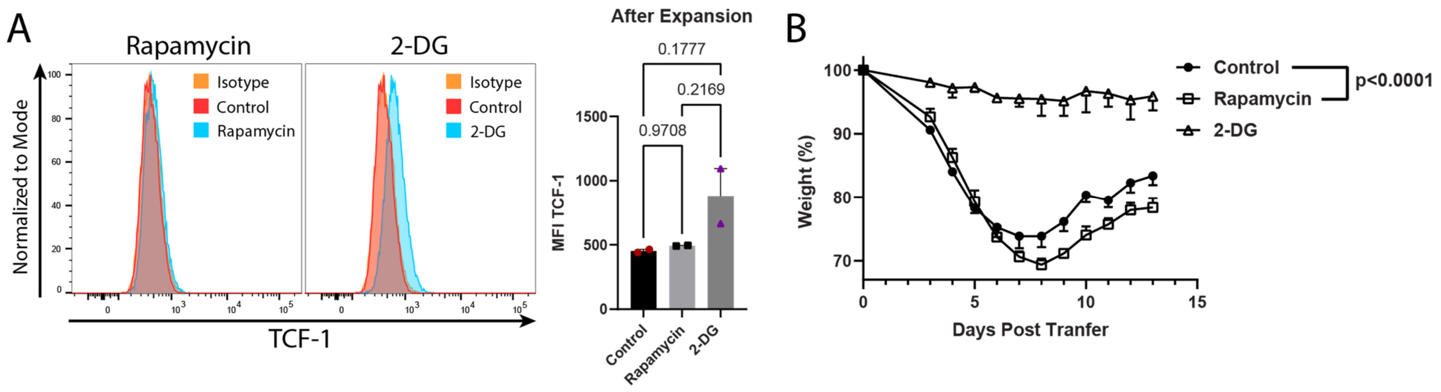

Supplemental Figure 5. Rapamycin-treated 8.8 CD8 T cells induce greater weight loss, and 2-DG-treated cells express TCF-1. A) Expression of TCF-1 among control, rapamycin- and 2-DG-treated CD8 T cells after 3-day stimulation and 3-day rest. Cells were treated with 4 mM 2-DG during both activation and rest. **B)** Change in weight of CD8-EAE mice after disease was induced by transfer of control, rapamycin- or 2-DG-treated CD8 T cells. Significance was determined by a two-way ANOVA comparing the control group with each treatment.

Supplemental Figure 6

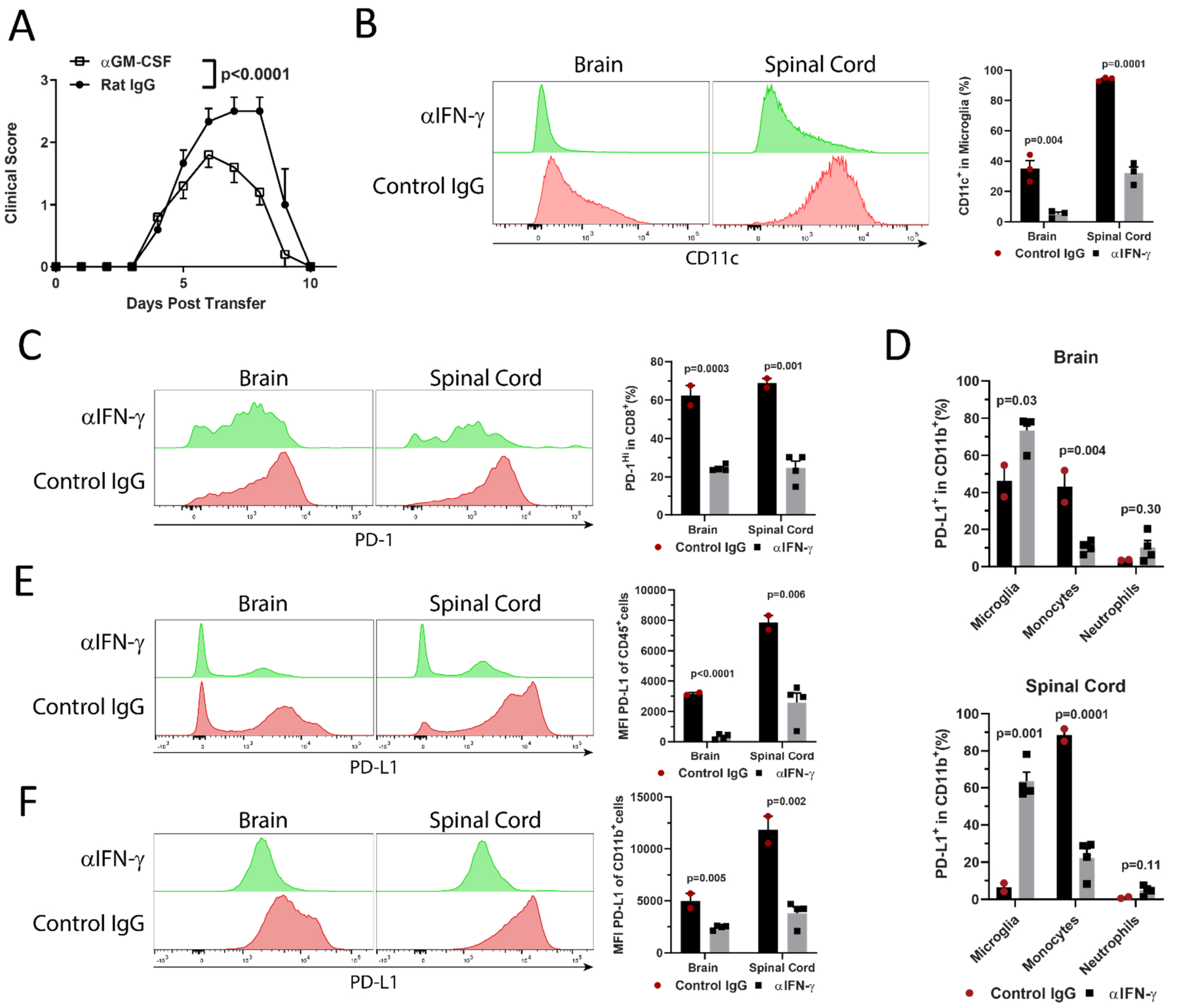

Supplemental Figure 6. Blocking GM-CSF suppresses EAE, and PD-L1 expression is altered by blocking IFN-γ. A) Clinical scores of CD8-EAE mice treated with anti-GM-CSF or isotype control mAb (400 μg/day; n=5-10 per/group). **B)** Histograms depicting expression of CD11c by microglia and the frequency of CD11c+ microglia in the brain and spinal cord of mice treated with anti-IFN-γ mAb or control IgG. **C)** Histograms depicting expression of PD-1 among CD8+ cells and the frequency of PD-1Hi CD8+ cells in the brain and spinal cord of mice treated with anti-IFN-γ mAb or control IgG. **D)** Distribution of myeloid cells that were PD-L1+CD11b+ in the brain and spinal cord of anti-IFN-γ– or isotype mAb-treated mice with CD8-EAE that were sacrificed on day 7 post-transfer. **E)** Histograms and quantification of PD-L1 MFI among CD45+ cells from mice treated with αIFN-γ or control IgG. **F)** Histograms depicting expression of PD-L1 among CD11b+ cells and the MFI of PD-L1 among CD11b+ cells in the brain and spinal cord of mice treated with anti-IFN-γ mAb or control IgG. Significance was determined by a t-test.
